# Betz cells of the primary motor cortex

**DOI:** 10.1002/cne.25567

**Published:** 2024-01-21

**Authors:** Matthew Nolan, Connor Scott, Patrick. R. Hof, Olaf Ansorge

**Affiliations:** ^1^ Nuffield Department of Clinical Neurosciences University of Oxford Oxford UK; ^2^ Department of Neurology Massachusetts General Hospital Boston Massachusetts USA; ^3^ Nash Family Department of Neuroscience and Friedman Brain Institute Icahn School of Medicine at Mount Sinai New York New York USA

**Keywords:** amyotrophic lateral sclerosis, Betz cell, motor cortex, neuroanatomy, projection neurons

## Abstract

Betz cells, named in honor of Volodymyr Betz (1834–1894), who described them as “giant pyramids” in the primary motor cortex of primates and other mammalian species, are layer V extratelencephalic projection (ETP) neurons that directly innervate α‐motoneurons of the brainstem and spinal cord. Despite their large volume and circumferential dendritic architecture, to date, no single molecular criterion has been established that unequivocally distinguishes adult Betz cells from other layer V ETP neurons. In primates, transcriptional signatures suggest the presence of at least two ETP neuron clusters that contain mature Betz cells; these are characterized by an abundance of axon guidance and oxidative phosphorylation transcripts. How neurodevelopmental programs drive the distinct positional and morphological features of Betz cells in humans remains unknown. Betz cells display a distinct biphasic firing pattern involving early cessation of firing followed by delayed sustained acceleration in spike frequency and magnitude. Few cell type‐specific transcripts and electrophysiological characteristics are conserved between rodent layer V ETP neurons of the motor cortex and primate Betz cells. This has implications for the modeling of disorders that affect the motor cortex in humans, such as amyotrophic lateral sclerosis (ALS). Perhaps vulnerability to ALS is linked to the evolution of neural networks for fine motor control reflected in the distinct morphomolecular architecture of the human motor cortex, including Betz cells. Here, we discuss histological, molecular, and functional data concerning the position of Betz cells in the emerging taxonomy of neurons across diverse species and their role in neurological disorders.

## Graphical abstract

Betz cells are ‘gigantopyramidal’ extratelencephalic projection neurons of the primary motor cortex that are part of the monosynaptic cortico‐motoneuronal pathway in humans and other primates. Here, we provide a comprehensive overview of the historic and emerging position of Betz cells in the evolutionary, functional and molecular taxonomy of neurons. We consider their role in neurological disorders and identify unanswered questions for research into the nature of these fascinating cells.

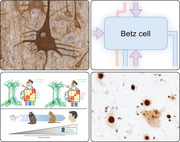

## 1 INTRODUCTION

**FIGURE 1:**
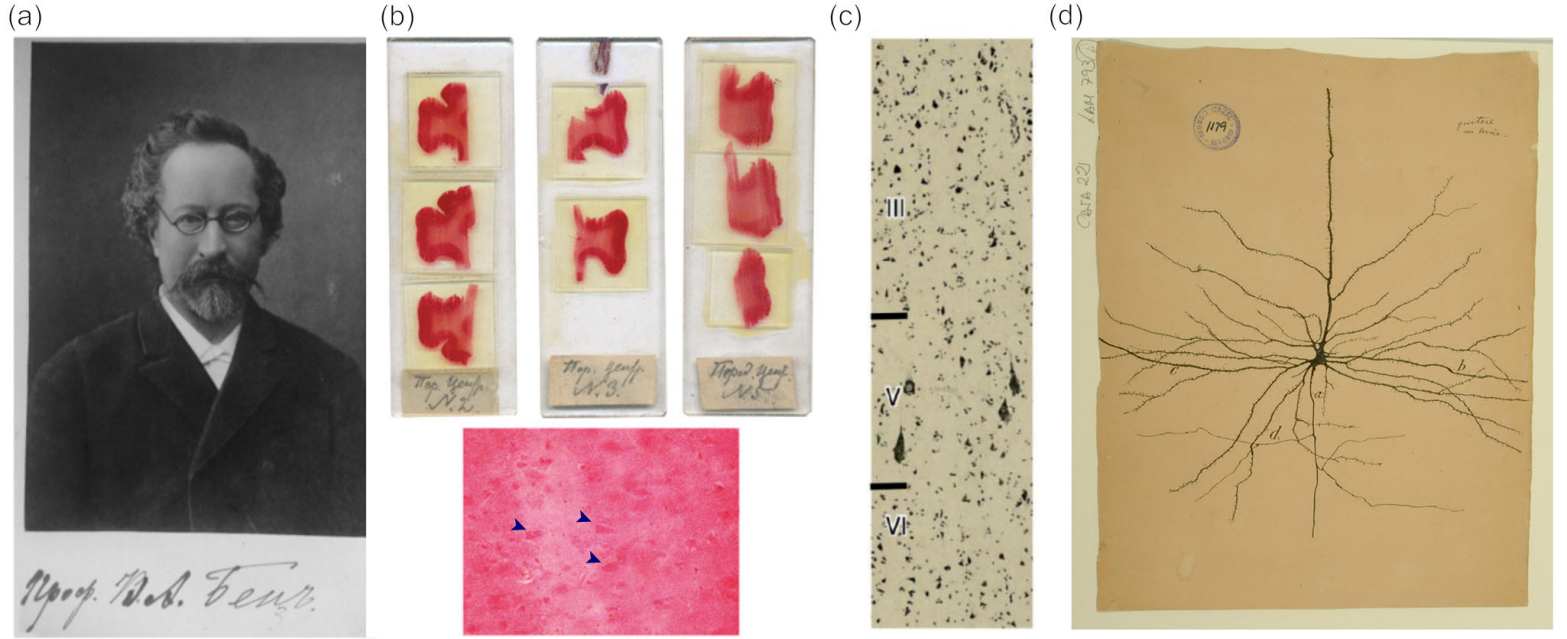
Betz cells in original preparations by Betz, Brodmann, and Ramòn y Cajal. (a) Portrait of Vladimir Betz with his Cyrillic signature in his self‐published Atlas of the Human Brain (1879). (b) Betz's original slide preparations stained with his brilliant carmine method. Arrowheads indicate the “Riesenpyramiden.” (c) Brodmann's original micrograph depicting the distinct layers of area 4 (primary motor cortex); note area 4‐defining Betz cells in layer Vb. (d) Original drawing by Ramòn y Cajal of a giant pyramid of the motor cortex. *Source*: Modified and reproduced with permission from the publisher: (a and b) Kushchayev et al. (2012); (c) Zilles (2018); (d) Courtesy of the Cajal Institute, Cajal Legacy, Spanish National Research Council (CSIC), Madrid, Spain.

Volodymyr Oleksiyovych Betz (1834–1894) was a Ukrainian neuroanatomist who published extensively on the cytoarchitecture of the cerebral cortex in the second half of the 19th century (Figure 1). Propelled by his own technological advances in microscopy, fixation, and staining, Betz made defining contributions in elucidating the microanatomy of the brain—at a time when the understanding of the relationship between neuroanatomical structure and functional organization was in its infancy—and built on the previous work of other preeminent neuroanatomists of the time including Broca, Meynert, Baillarger, and Campbell, who collectively laid the foundations for future understanding of functional neuroanatomy. Betz collected and studied nearly 9000 brains from various species (Kushchayev et al., 2012). Betz (1874) made perhaps his most famous contribution through his description of the “cumulative nests” of “giant pyramids” (*Riesenpyramiden*) of the precentral gyrus in humans, which he originally described as being located in Meynert's layer IV. Betz's observations were considered by Lewis and Clarke in their study of cortical lamination of the motor area, where they refer to “the nests of Betz” when discussing the distinct clustering of the “giant pyramids” (Bevan‐Lewis, 1878). This is probably the first instance Betz's name was associated with these cells in the English scientific literature. Subsequent revisions of cortical layer terminology (by Brodmann) identified these “giant pyramids” as being in layer Vb (Figure 1c).

**FIGURE 2:**
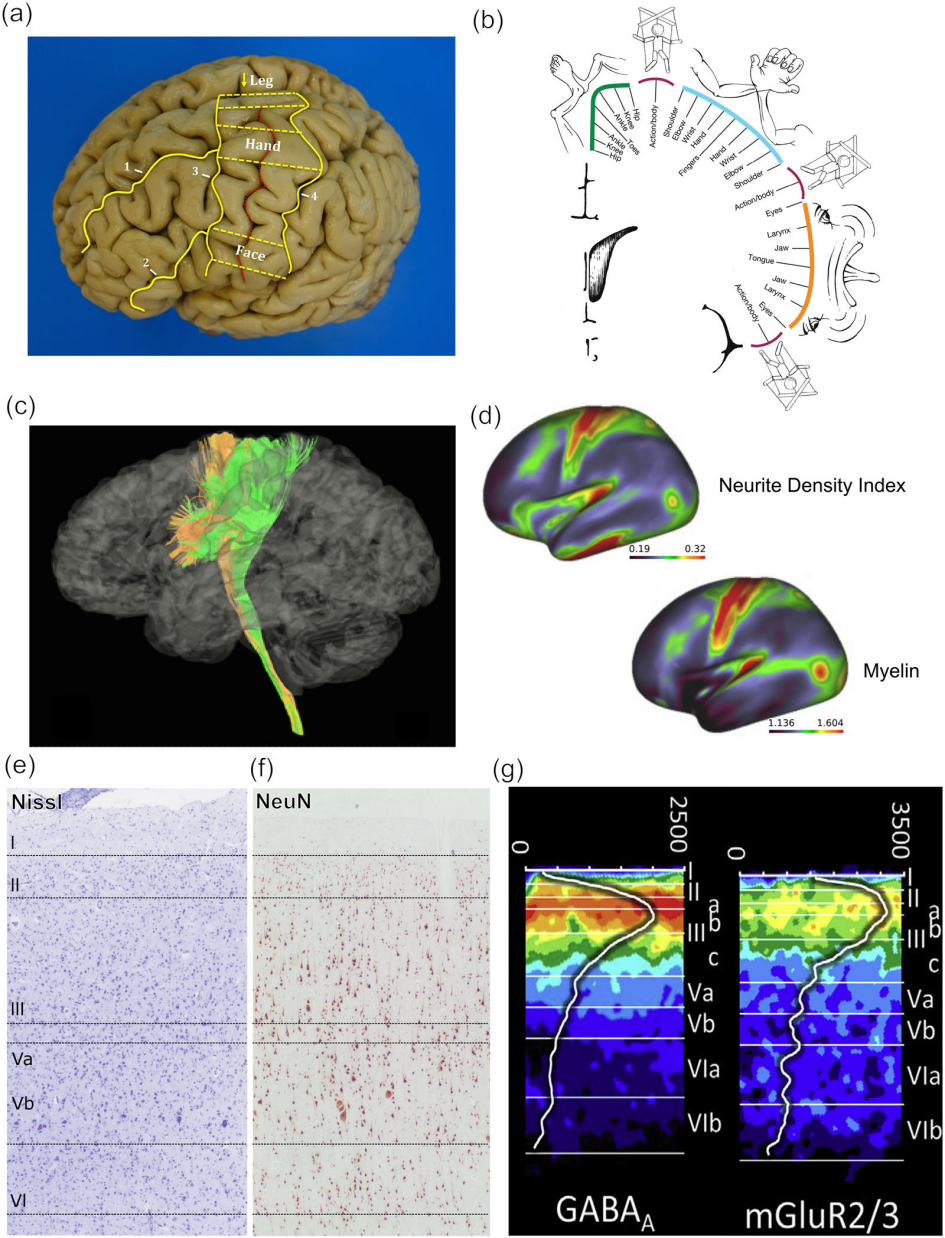
Anatomy, cyto‐, and chemoarchitecture of the human primary motor cortex. (a) Landmarks that aid macroscopic identification of the human primary motor and sensory cortex in a formaldehyde‐fixed brain. However, note that cytoarchitecturally defined area 4 (e and f) is mostly hidden in the depth of the central sulcus on the posterior bank of the precentral gyrus, and only partially present on its crest (1, superior frontal sulcus; 2, inferior frontal sulcus; 3, precentral sulcus; 4, postcentral sulcus; the red line marks the trajectory of the Rolandic fissure). (b) Illustration of the recently proposed “integrate‐isolate” concept of topographical organization of the human motor cortex (Gordon et al., 2023). This model suggests that classical (homunculus) effector zones (foot (green), hand (cyan), mouth (orange)) alternate with zones that do not elicit distinct movements but reflect M1 connectivity with cingulo‐opercular structures involved in motor planning and control of complex whole‐body movements (magenta). (c) Axons from extratelencephalic projection (ETP) neurons, including Betz cells, converge to form the corticospinal tract. Depicted are the fibers arising from area 4 (green) and area 6 (orange). (d) Diffusion magnetic resonance imaging (MRI)‐derived neurite and myelin properties averaged from 505 adults. Area 4 is characterized by a very high neurite density index and myelin content (red). (e and f) Cytoarchitecture of human primary motor cortex depicted with Nissl histochemistry and NeuN immunohistochemistry. Note Betz cells in layer V, including a cluster of three Betz cell somata highlighted by NeuN stain in (f). (g) Chemoarchitecture of human area 4 revealed by GABA_A_ and mGluR2/3 receptor subunit autoradiography (pseudocolored: gradients from blue to red indicate low‐to‐high binding across cortical layers); the white profile curve overlaid on the autoradiograph images indicates the absolute concentrations (in fmol/mg protein) of GABA_A_ and mGluR2/3, respectively. *Source*: Figures distributed under the terms of the CC BY 4.0 license: (a) Pallebage‐Gamarallage et al. (2018), (b) Gordon et al. (2023), (c) Wang et al. (2019), (d) Fukutomi et al. (2018), and (g) Palomero‐Gallagher and Zilles (2019).

Betz cells are primarily distinguished morphologically from neighboring pyramidal cells through their large size, accumulation of intracellular lipofuscin, and circumferential or complex basal dendritic architecture (Braak & Braak, 1976; Jacobs et al., 2018; Meyer, 1987; Sherwood et al., 2003; Szocsics et al., 2021). In the strictest sense, “Betz cell” refers to the gigantopyramidal neuron of the human primary motor cortex. However, Betz himself recognized the “giant pyramids” also in other primates and other mammals. In humans, Betz cells represent only a minority of layer V extratelencephalic projection (ETP) neurons. Although input from the primary motor cortex contributes around 30% of fibers to the corticospinal tract, Betz cells comprise only around 10% of pyramidal neurons in layer V of the primary motor cortex (Rivara et al., 2003) and around 2%–3% of so‐called pyramidal tract neurons (Lassek, 1940). The primary projection of Betz cell axons is to the corticospinal tract (Figure 2c), most of which crosses at the level of the medullary pyramids before its axons terminate at specific levels in the gray matter of the contralateral spinal cord, including anterior horns. It is unknown if human Betz cells exclusively innervate monosynaptically α‐motoneurons in Rexed lamina IX of the spinal gray matter (as is often assumed). At least in the macaque, there are dense corticospinal projections to neurons beyond Rexed lamina IX, into laminae VIII and VII (Morecraft et al., 2013). Whether any of these projections arise from bona fide Betz cells remains unknown; in the absence of a Betz cell‐specific cytoplasmic (including axonal) marker, their collateral and terminal axonal ramifications remain obscure.

Historically, and in clinical parlance, the Betz cell is considered the quintessential “upper motoneuron” (or “pyramidal tract neuron”) presumed to be selectively vulnerable in amyotrophic lateral sclerosis (ALS) and hereditary spastic paraplegia (HSP). However, Betz cells do not share typical neurophysiological or transcriptome characteristics of true “motoneurons” of the brainstem or Rexed lamina IX (Lemon, 2021); therefore, they are best referred to as giant pyramidal neurons belonging to the class of ETP neurons of the primary motor cortex (as intended by Betz). Their absence in rodents suggests that experimental insights gained from studies of layer V corticospinal projection neurons in mice—which, unlike Betz cells, do not connect monosynaptically with spinal α‐motoneurons motoneurons—may be of limited translational value for our understanding of ALS and HSP pathogenesis (Genc et al., 2019; Lemon, 2019). Perhaps, human vulnerability to ALS reflects our uniquely evolved corticospinal motor circuitry, in which Betz cells form an essential component (Lemon, 2019, 2021).

One of the challenges—and opportunities—of contemporary neuroscience and neuroanatomy is to reconcile historical observations and concepts with insights derived from the avalanche of functional and novel single‐cell or single‐nuclei “multiomic” studies (Miller et al., 2020; Yuste et al., 2020): How do we arrive at a consistent terminology for the diverse neuronal types of the human brain? Is a taxonomy of the “ground truth” even possible or desirable, given the cellular plasticity of the nervous system across the life span and its functional organization as dynamic microscopic and macroscopic cellular networks? Here, we attempt to answer some of these questions pertaining to the giant pyramids of Betz, which have intrigued students of the human brain for well over 100 years. For the purposes of this review, we define a Betz cell as a distinct morphofunctional unit of the primary motor cortex in primates that makes monosynaptic contact with brainstem and spinal α‐motoneurons and belongs to the broad transcriptional class of layer V ETP neurons. Our definition is in line with that adopted by Jacobs et al. (2018), who apply the term “Betz cell” to the gigantopyramidal neurons in the motor cortex of primates, not other mammals. We acknowledge that a precise taxonomy using historical terms (such as “Betz cell”) will be replaced by emerging multidimensional definitions of neuronal classes, as new data from primate and non‐primate brains are integrated (Miller et al., 2020). However, we—and others engaged in molecular analysis of the motor cortex—believe the usage of the term “Betz cell,” as defined here, remains justified as a concept of a morphofunctional neuronal unit until more comprehensive data across the animal kingdom become available. We also suggest that the term “ETP neuron” is an appropriate operational definition (and abbreviation) for the transcriptional class that Betz cells belong to. This is in line with established terminology, for example, Bakken et al. (2021; Table 1). The purpose of our article is to review and discuss historical (Walshe, 1942) and current knowledge about the nature of the “Betz cell” in evolution, health, and disease, to inform future studies that may settle the precise designation of these fascinating cells in the taxonomy of neurons.

**TABLE 1:** Betz cell transcriptomic terminology.

Transcriptomic cluster	Isolation method	Classification method	Validation method	Limitations	Drawbacks	Study
BCL11B/EYA4 (FEZF2+ CRYM+ subtype) BCL11B/THSD4 (FEZF2+ CRYM+ subtype)	snRNA‐seq (unbiased)	ACTIONet with curated list of cell‐specific markers	Immunofluorescence (CRYM)	Betz cells identified in silico using L5 neuronal markers rather than Betz cell specific markers RNA content (a proxy of cell size) was used to identify Betz cells from other L5 neurons	Conflicting reports of Betz cells expressing CRYM Betz cell size continuum overlaps with other L5 neurons	Pineda et al. (2021)
L5 FEZF2 CSN1S1 L3‐5 FEZF2 ASGR2	snRNA‐seq (unbiased)	Seurat with unbiased marker discovery	In situ hybridization of L5 ET markers with Betz cell enriched marker (NEFH) PATCH‐seq (macaque and human)	Lack of Betz cell‐specific markers limits ISH validation to L5 ET markers (POU3F1, GRIN3A, SERPINE2) PATCH‐seq validated using presumed Betz cell from human premotor cortex	No Betz cell‐specific cluster identified Cluster also contains non‐Betz cell ET neurons	Bakken et al. (2021)

*Note*: Summary of human Betz cell clusters and their terminology previously identified in snRNA‐seq studies. See main text for details.

## 2 THE PRIMARY MOTOR CORTEX

### 2.1 Structural considerations

The primary motor cortex in mammals, including humans, is in the frontal lobe, immediately anterior to the primary somatosensory cortex, separated by the Rolandic (or central) sulcus, or primary fissure in gyrencephalic species (Figure 2a). It contains a complete map of the body's neuromuscular system. Its precise borders are defined cytoarchitecturally; however, although macroscopic landmarks can be used to identify it with reasonable certainty, the assumption that the macroscopic gyral pattern can be used to define the precise cytoarchitecturally defined location of “Brodmann's area 4” (hereafter called area 4) in an individual brain is wrong (Rademacher et al., 2001). Generally, its posterior border with the primary somatosensory cortex maps to the fundus of the central sulcus. Other landmarks, such as the “inverted omega sign” (or “hand knob”), are commonly used in clinical magnetic resonance imaging (MRI) to locate the presumed cortical representation of the hand and fingers (Yousry et al., 1997). However, even Penfield and Boldrey's (1937) seminal study on the somatotopy of the functional surface map of the human primary motor cortex suggests that somatotopic boundaries are more overlapping than generally depicted in the classical “homunculus” (Catani, 2017). Specifically, recent functional studies in humans suggest that one may be able to divide the primary motor cortex into macroscopically distinct zones that reflect the classical (concentric) effector somatotopies of the homunculus (foot, hand, and mouth movements) alternating with areas (inter‐effector regions) that do not elicit distinct movements but are recruited for complex whole‐body action planning with the cingulo‐opercular network (Gordon et al., 2023; Figure 2b). The motor cortex in humans generally shows a high degree of interhemispheric symmetry; however, neuropil volume of the hand region and the precentral component of the pyramidal tract is larger in the left hemisphere of the majority of postmortem brains, consistent with there being a majority of right‐handed people (Amunts et al., 2000; Rademacher et al., 2001; Volkmann et al., 1998).

The integration of macroscopic, microscopic, and functional definitions of cortical areas using unbiased and probabilistic in vivo and ex vivo approaches allows further refinement of how cerebral structure relates to function (Amunts & Zilles, 2015; Rademacher et al., 2001; Rapan et al., 2021). In this context, it is interesting to note that the overall definition of the cytoarchitectonic borders of the primary motor cortex (area 4), as defined by Brodmann, remains generally accepted, despite the discovery of novel subdivisions within it (Rapan et al., 2021). Indeed, the primary motor cortex can be defined by the presence of Betz cells in layer Vb (Figures 1c and 2e,f), and Brodmann considered them to be the only specific type of neurons of the human cerebral cortex that can be used to define a unique area cytoarchitecturally (Zilles, 2018). All other areas require a combination of more than one cytoarchitectural feature for identification. Another distinctive—but not specific—feature of the adult human primary motor cortex is its deviation from the general six‐layer isocortical organization: Layer IV is not well developed and area 4 has therefore been referred to as an “agranular” cortex. However, whether or not the human primary motor cortex lacks layer IV neurons remains controversial and is an example of the challenges of arriving at a “definitive” spatially and temporally resolved taxonomy of neurons, as morphological and transcriptomic identities may not overlap perfectly, nor remain static (Amunts et al., 1997; Armananzas & Ascoli, 2015; Bakken et al., 2021; Barbas et al., 2015; García‐Cabezas & Barbas, 2014; Yamawaki et al., 2014; Zeng & Sanes, 2017). Historically, Santiago Ramón y Cajal recognized a rudimentary layer IV in the human primary motor cortex, which was disputed by Brodmann who claimed that granular layer IV was present only transiently during development but absent in adult human area 4 (García‐Cabezas & Barbas, 2014). Interestingly, the persistence of a granular layer IV in the human motor cortex may be a feature of certain neurodevelopmental disorders (Amunts et al., 1997). Recent layer‐enriched single nuclei transcriptome analysis of human primary motor cortex and comparison with data sets from human middle temporal gyrus (which contains a well‐defined layer IV) suggests that layer IV‐like glutamatergic neurons corresponding to the “ESR1 type” of mid‐temporal gyrus layer IV neurons are indeed present in human area 4, albeit sparsely (Bakken et al., 2021). Other characteristics of the primary motor cortex (compared to its posterior border with area 3a) are a wide band of gray matter, pronounced columnar arrangement of cells, and a relatively indistinct boundary with subcortical white matter, which can make assessments of cortical thickness in health and disease difficult (la Fougere et al., 2011; Rademacher et al., 2001). Finally, it is important to note that most of the cytoarchitecturally defined area 4 is restricted to the posterior bank of the precentral gyrus, in the depth of the central sulcus. The classical depiction of the projection of area 4 on to the surface (crown) of the precentral gyrus is therefore misleading (Rademacher et al., 2001) but nevertheless useful for clinical purposes.

The anterior border between the primary motor cortex and the premotor cortex is not defined by a macroscopic landmark like the precentral or any other sulcus and requires cytoarchitectural criteria for distinction (Amunts & Zilles, 2015; Ruan et al., 2018). It has been proposed that the supplementary motor area on the medial surface of the brain can be distinguished from the primary motor cortex by a transition to densely packed, large pyramidal cells in the lower parts of layer III and cessation of Betz cells in layer V (Ruan et al., 2018). However, a recent study of a neurosurgical resection specimen claims that “Betz‐like” cells are also found in what can be macroscopically defined as the human premotor cortex (Bakken et al., 2021), consistent with previous observations in macaques (Vigneswaran et al., 2011). A better appreciation of the parcellation of the primary motor cortex can be achieved with modern experimental approaches that integrate cyto‐, myelo‐, and neurotransmitter receptor‐based architecture (Palomero‐Gallagher & Zilles, 2019; Figure 2g) or employ unbiased methods (Rademacher et al., 2001; Schleicher & Zilles, 1990). The gray level index, a measure of quantitative cytoarchitecture, can be used to map cortical layer structure in regions that are otherwise ill‐defined using a combination of cell body fraction volumes, cell size, and density (Schleicher & Zilles, 1990). The primary motor cortex has a distinctly low gray level index value that indicates low cell body fraction volume and larger space among cell bodies than other cortical areas (Amunts et al., 2007), which seems to be associated with high neurite density and cortical myelin content (Fukutomi et al., 2018; Figure 2d), implying a greater density of (fast) synaptic connections between the relatively sparsely distributed neurons. How the postulated differences in cortical thickness, fractional anisotropy, and intracortical myelin content between somatotopic effector areas and inter‐effector regions described using MRI metrics in vivo (Gordon et al., 2023; Figure 2b) may be reflected cytoarchitectonically remains to be studied.

### 2.2 Functional considerations

The primary motor cortex can be represented based on the extent of its involvement and association with particular muscle groups (Catani, 2017; Penfield & Boldrey, 1937), with larger representations such as hand and face signifying higher proportions of cortical involvement and complexity of encoded movements, consistent with the evolution of distinctly human skills of refined finger movements and speech (Figure 2a). However, it is not entirely cleanly defined somatotopically, and there is notable representational overlap between areas (Catani, 2017; Rathelot & Strick, 2006; Schieber & Hibbard, 1993, Figure 2b). Studies in monkeys and humans indicate that there are overlapping “colonies” of corticospinal projection neurons in the primary motor cortex, particularly concerning innervation of muscles of the arm, wrist, and digits (Catani, 2017; Dancause, 2013; Schieber & Hibbard, 1993; Silverstein, 2012). Retrograde tracer studies in rhesus monkeys indicate that there is no evidence for focal clustering of individual layer V projection neurons that connect monosynaptically to α‐motoneurons innervating a specific hand muscle (Rathelot & Strick, 2006). This study also confirmed that both gigantopyramidal and smaller layer V pyramidal neurons project monosynaptically to α‐motoneurons serving a specific hand muscle. Functionally, this is evidenced by intracortical stimulation of specific sites within the motor cortex eliciting a response from multiple muscle groups, and individual muscles can be stimulated at several sites (Schieber & Hibbard, 1993; Silverstein, 2012). However, the artificial nature of intracortical stimulation bears little resemblance to the small‐scale (microscopic) localized pattern of neuronal activity that regulates natural (volitional) movement. Further, some of the described data stems from nonhuman primates, who have distinctively less fine motor control of digits; therefore, the degree of representational overlap between functions of cortical areas may be different in humans. There is still some debate, therefore, surrounding whether the primary motor cortex best represents individual muscle groups, or “movements” of multiple groups, and whether the stimulation of the primary motor cortex elicits or suppresses movement (Ebbesen & Brecht, 2017). The idea that “stimulation” of motor cortical areas results in downstream “inhibition” (as well as “activation”) of select targets to facilitate the execution of complex, finely tuned volitional movements, is often not appreciated (Ebbesen & Brecht, 2017).

Perhaps the best somatotopical resolution of motor cortex representation in humans has been defined for fine finger movements. Functional MRI studies at 7 T using Gaussian population receptive fields modeling found that the thumb representation is located ventrolaterally near the hand area at the crown and posterior bank of the precentral gyrus, while representations of the remaining four finger digits gradually shift in the dorsomedial direction along the precentral gyrus (Schellekens et al., 2018). Again, this particular pattern seems to relate to a specific function, namely, finger digit flexion and not finger digit extension, and a pattern of activity suggesting the integration of primary motor and primary sensory signals for individual digits (Schellekens et al., 2018). However, any such conclusions about individual digit control must be interpreted in the context of the applied methods and their resolution (Arbuckle et al., 2020), and the fact that isolated movements (single finger flexion/extension) in a laboratory setting may not reflect cortical network activity occurring during complex natural (goal‐directed) movements, which clearly involve motor control of the whole body (e.g., positioning, breathing, and arousal). Direct recordings from the primary motor cortex in nonhuman primates suggest that the structural principles of neuronal pattern generation are different for arm (reach) and hand (grasp) control (Suresh et al., 2020). In summary, it should be recognized that execution and inhibition of volitional (planned) movement is dependent on complex network activity, involving diverse areas of the frontal lobe anterior to the anatomically defined “primary” motor cortex and that the idea of a static homunculus confined to the cytoarchitecturally defined cortical boundaries of area 4 is an oversimplification.

**FIGURE 3:**
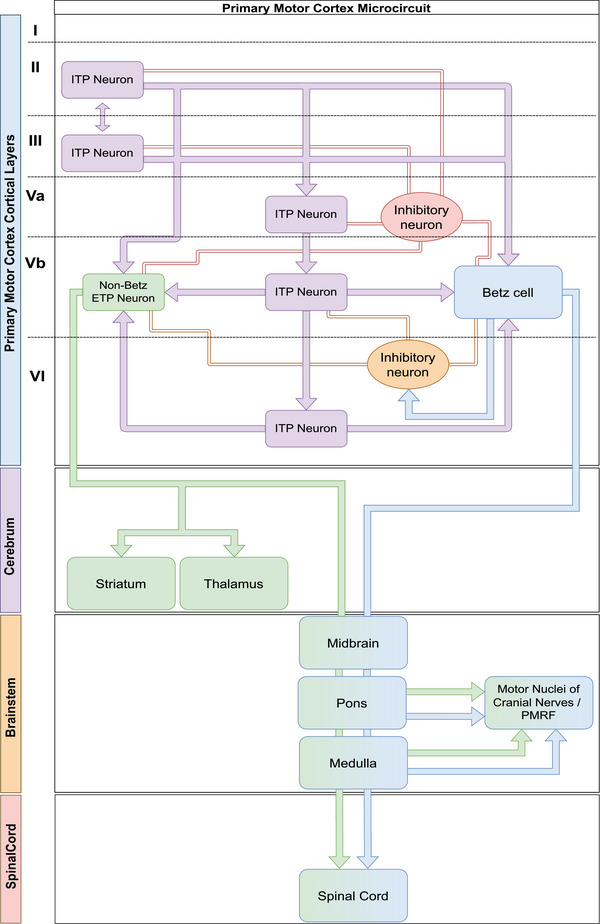
Putative position of the Betz cell in human area 4 microcircuitry. Previously published diagrammatic depictions of area 4 microcircuitry generally do not distinguish Betz cells from other layer V extratelencephalic projection (ETP) neurons. Here we integrate the Betz cell in a putative schematic of area 4 microcircuitry using information from McColgan et al. (2020) and Bakken et al. (2021). The precise relationship between bona fide Betz cells, as defined in this review, and other non‐Betz layer V ETP neurons, some of which do send collaterals to intratelencephalic targets (e.g., the striatum), remains to be defined. To the best of our knowledge, intratelencephalic targets of primate Betz cells have not been identified. The schematic has been greatly simplified to emphasize that Betz‐ and non‐Betz types of layer V ETP neurons should be considered in any future modeling of human‐specific motor circuitry. Interneuron and other cellular diversity and connectivity of primate area 4 are therefore not fully represented here. It is also acknowledged that not all motor nuclei of the cranial nerves receive direct monosynaptic Betz cell innervation; motoneurons controlling eye movements may only receive indirect input. Generic drawing for brainstem connectivity was chosen to avoid complexity; this includes the omission of the pyramidal decussation of the majority of the pyramidal tract (and therefore, ETP neuron axons). ITP, intratelencephalic projection; PMRF, pontomedullary reticular formation.

How these observations relate to layer‐ and cell‐specific neuronal connectivity within and beyond anatomically defined area 4 in humans remains unclear. A complete “wiring” diagram of the human primary motor cortex does not exist. Here, we provide a sketch of a putative area 4 intracortical neuronal network (Figure 3) based on recent data and concepts (Bakken et al., 2021; McColgan et al., 2020). Interestingly, most published diagrams of layer V ETP neurons of the primary motor cortex (sometimes also referred to as pyramidal tract neurons; Gerfen et al., 2018; Harris & Shepherd, 2015) do not attempt to distinguish between Betz‐ and non‐Betz layer V pyramidal cells, leaving open the question concerning their molecular and functional relationships and roles in the motor circuitry (Gerfen et al., 2018; Harris & Shepherd, 2015; McColgan et al., 2020; Sahni et al., 2020).

## 3 DISTRIBUTION AND MORPHOLOGY OF HUMAN BETZ CELLS

### 3.1 Distribution

**FIGURE 4:**
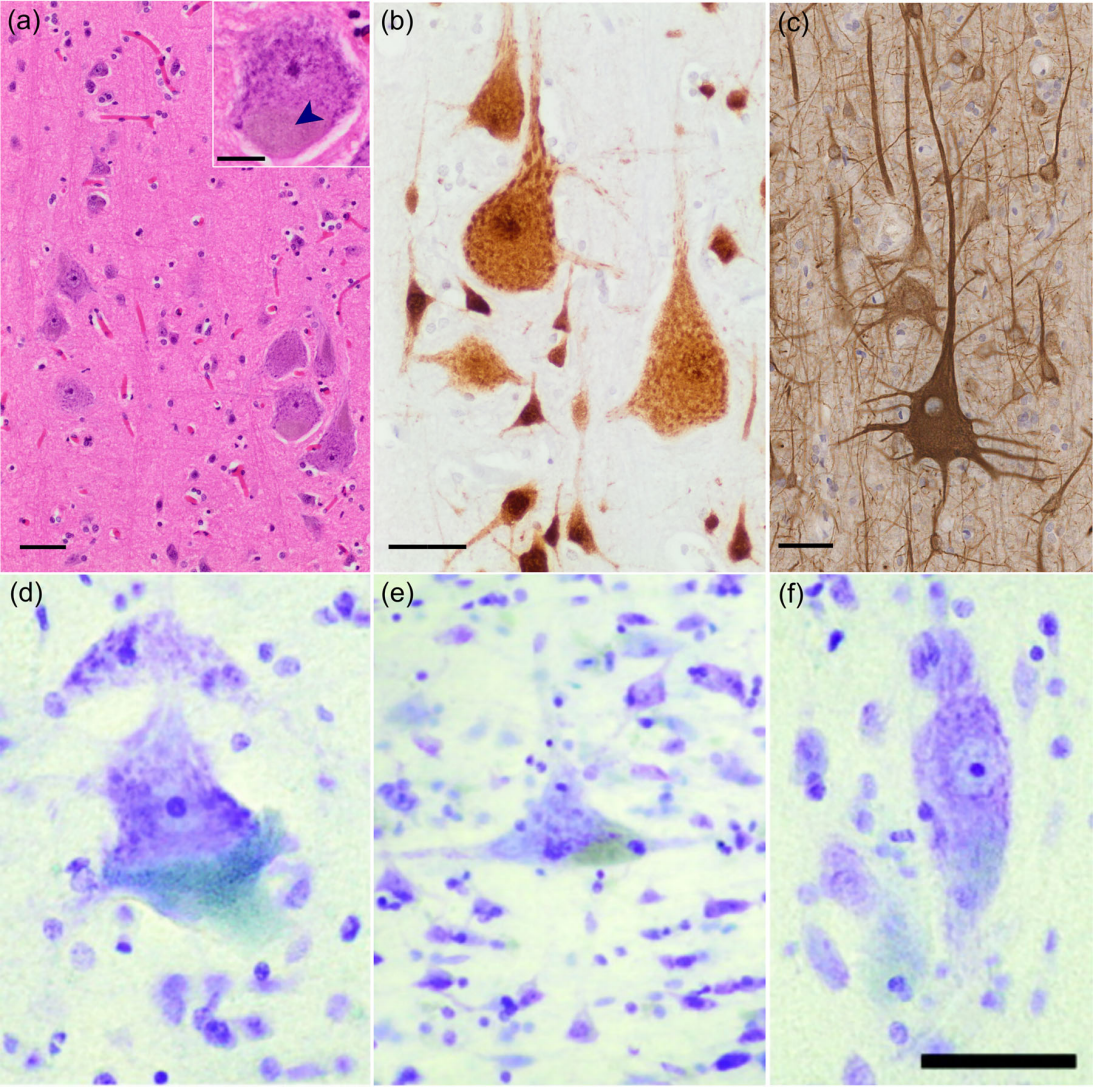
Morphology of human Betz cells in the primary motor cortex. Columnar nests of Betz cells are easily identifiable in layer Vb of the leg area of the human primary motor cortex (a, hematoxylin and eosin stain). Here, Betz cell somata are round or polygonal, they contain coarse rough endoplasmic reticulum and abundant “packaged” lipofuscin granules at one (often basal) pole of the soma (a, inset, arrow). They express the transcription factor FOX‐3 (anti‐NeuN antibody, b), and their soma and distinctive circumferential dendritic arborization can be highlighted with antibodies against non‐phosphorylated neurofilaments (SMI 311, c). Betz cells exhibit morphological variability across the length of primary motor cortex: Near the border of area 4/area 3 Betz cells are rounded or squatted (d); in the vicinity of the “hand‐knob” region they appear more triangular (e); while at the rostral boundary between the primary motor cortex and the border of area 6, Betz cells exhibit a more fusiform shape (f). Scale bars: 50 μm. The blue–green granular signal in (d) and (e) represents the basal lipofuscin aggregates. *Source*: Parts (d–f) were reproduced and modified with permissions from Rivara et al. (2003).

Betz cells are the largest neurons in the human CNS, with axonal projections spanning up to 1 m or more in humans. Early estimates of total Betz cell number ranged from 25,000 to 40,000 per hemisphere (Campbell, 1904; Lassek, 1940; Scheibel & Scheibel, 1978; Scheibel et al., 1977); however, a more recent stereological study of six human brains found a mean total of 125,290, albeit with significant inter‐case variability, and accounting for around 10% of pyramidal neurons in layer Vb (Rivara et al., 2003). In the mature human brain, they occur in columnar clusters of a few cells (Figure 4a,b). Retrograde tracer studies in nonhuman primates indicate that these Betz cell “nests” are mirrored in each hemisphere and project to the same spinal level (Groos et al., 1978). This is likely of functional relevance (Bundy & Leuthardt, 2019). To our knowledge, there is no evidence of direct transcallosal projections of Betz cell axons to the contralateral hemisphere. How Betz cells within and between clusters from the same hemisphere relate to each other genetically or functionally is also unknown. Betz cell distribution follows a mediolateral gradient around the precentral gyrus; the most densely clustered Betz cell nests reside in a zone midway from the midline along the mediolateral axis, and the largest Betz cells are found in the foot and leg region of the homunculus on the most medial part of area 4 (Rivara et al., 2003). Betz cells decrease in both size and number moving ventrolaterally along the central sulcus (Meyer, 1987), with around 75% of all Betz cells present in the upper third segment of area 4 (Lassek, 1940; Rivara et al., 2003).

Stereological estimations of human Betz cell soma size vary. Betz himself described their average size as 60 × 120 μm^2^ (Betz, 1874), Brodmann reported them as 53 × 106 μm^2^ (Brodmann, 1909; Brodmann & Garey, 2006), whereas Rivara et al. (2003) recorded an average cell body volume of 86,685 μm^3^. However, as noted above, there is considerable variation in the size and shape of the cell body of Betz cells (Figure 4). Early theories suggested that Betz cell soma size was proportional to either axonal length or the size of the dependent muscle, as the largest Betz cells are present in the cortical region represented by the lower limb areas of the homunculus (Rivara et al., 2003). In humans, although soma size and axonal length increase concomitantly with height, there is no relationship with body weight (Ho et al., 1992).

### 3.2 Morphology

Betz cells are commonly identified histologically by their size and presence in layer Vb of the primary motor cortex (Figures 1, 2, and 4). Although they are the largest cell type in this region, other large pyramidal neurons also exist in this layer whose size overlaps with the smallest Betz cells, making accurate visual identification problematic (Walshe, 1942), and as such size and location alone cannot be used to identify Betz cells from surrounding pyramidal populations (Lashley & Clark, 1946; Mettler, 1944). In contrast to the scattered deposition of lipofuscin granules seen in most neurons, mature Betz cells harbor large, well‐circumscribed lipofuscin agglomerations confined to one (usually basal) pole of the cell (Braak, 1976, Figure 4a,d,e). This “pigmento‐architectonic” approach combined with Golgi impregnation allowed the identification of a small gigantopyramidal field in the depth of the cingulate gyrus, which may represent evolutionary precursors of the Betz cells in area 4 (Braak, 1976; Braak & Braak, 1976).

However, it is their unique dendritic architecture that best distinguishes Betz cells from other layer V pyramidal neurons (Rivara et al., 2003). Unusually for neurons, and unlike neighboring pyramidal cells, Betz cells possess dendrites that arise around the full circumference of the soma (Figure 4c); in humans, these dendrites are not symmetric, are mostly aligned parallel to the main axis of the gyrus, and form a dense array of basilar dendrites (Meyer, 1987; Figure 4c). Betz cells also occasionally form long taproot dendrites that descend deep into the white matter (Scheibel et al., 1977) a feature found in multiple primates, felids, and canids (Deschenes et al., 1979; Jacobs et al., 2018; Nguyen et al., 2020). A small number of Betz cells appear to be bipolar, with basal dendrites entering the white matter (Meyer, 1987), but most have one long or bifurcated apical dendrite that can extend into the superficial layers (Braak, 1976; Meyer, 1987). Additionally, all Betz cell dendrites exhibit numerous spiny appendages and bulbous protrusions surrounding the soma (Braak & Braak, 1976). However, there is both interregional and interindividual dendritic variation, with dendritic asymmetry particularly evident in the transitional area between the precentral gyrus and central sulcus (Meyer, 1987).

The main projection of Betz cells is to the corticospinal tract, which (mostly) terminates in the anterior horn of the spinal cord, making monosynaptic contact with α‐motoneurons that in turn innervate target muscle groups to facilitate movement (Scheibel et al., 1974). Betz cells maintain some of the longest corticofugal axons in the human central nervous system, extending in adults for ∼60–70 cm from the paracentral lobule to the lumbar enlargement of the spinal cord. Betz cells only contribute 2%–3% of total axons in the primate corticospinal tract (Lassek, 1940; Lassek & Rasmussen, 1939). A recent study of corticofugal axonal projections in macaque monkeys originating in motor, premotor, and somatosensory cortices confirmed that by far the thickest axons originate in the primary motor cortex (Innocenti et al., 2019). The scarcity of thick axons within the corticospinal tract would therefore be consistent with an origin from Betz cells (Kaiserman‐Abramof & Peters, 1972; Sherwood et al., 2003; Terao et al., 1994). However, precise estimation of axon diameters, identification of parent cell bodies, and calculation of associated conduction velocities of pyramidal tract neurons originating in the primary motor cortex currently remain largely speculative, as there is no Betz cell‐specific cytoplasmic marker that would allow visualization of their axonal projections in human material (Kraskov et al., 2019).

## 4 EVOLUTIONARY BIOLOGY AND COMPARATIVE ANATOMY OF BETZ CELLS

**FIGURE 5:**
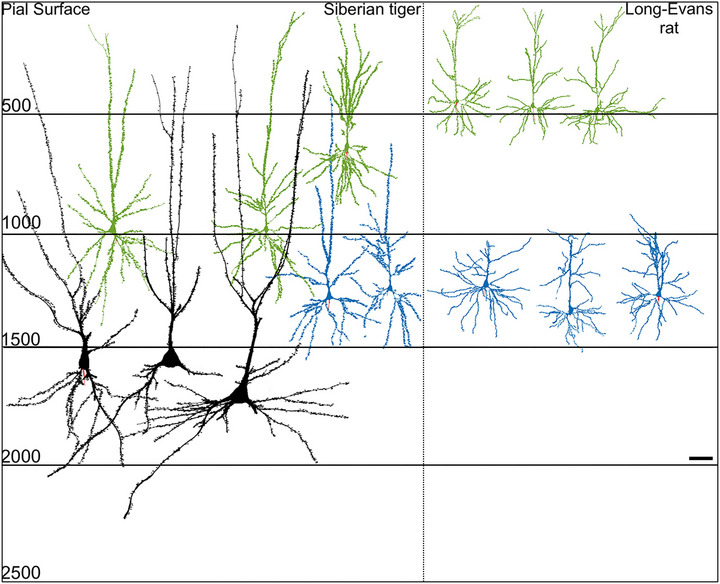
Gigantopyramidal neurons of the motor cortex in other mammals. Gigantopyramidal neurons are present in some non‐primate mammals, such as in large carnivores, where they are particularly large (Siberian tiger, left). By contrast, rodents, frequently used to study motor neuron disorders, do not possess bona fide gigantopyramidal neurons (Long‐Evans rat, right), nor a monosynaptic corticospinal pathway from layer V ETP neurons to α‐motoneurons. Neurolucida drawings: black—gigantopyramidal neurons; green—superficial pyramidal neurons; blue—deep pyramidal neurons. Left‐hand scale: distance from pial surface in micrometers (μm), scale bar: 100 μm. *Source*: Reproduced and modified with permissions from Jacobs et al. (2018).

The lack of access to appropriately sampled human postmortem or biopsy material has limited our understanding of Betz cell biology; much of our understanding of the molecular development and neuronal subtype specification of pyramidal neurons in the motor cortex has come from mouse modeling. The comparative and evolutionary anatomy of the motor cortex suggests that the molecular mechanisms that drive corticospinal motoneuron differentiation are unlikely to be identical between mice and humans; indeed, the expressions of only a few cell‐type‐specific genes are conserved across these species (Bakken et al., 2021). As noted above, rodents do not possess Betz cells as defined in this review and most of the literature (Figure 5). Further, although the anatomical organization of the corticospinal tract from the cortex to the brainstem is largely similar across mammalian species, there are distinct differences distal of the pyramidal decussation (Welniarz et al., 2017). Unlike in adult primates, the crossed portion of the corticospinal tract in mice descends in the dorsal spinal cord, and direct comparisons of the macaque, chimpanzee, and human corticospinal tract show that the Betz cells of these primates directly synapse with spinal motoneurons (Kuypers, 1958), while mature rodents do not possess such monosynaptic connections (Alstermark et al., 2004; Yang & Lemon, 2003), nor do they possess gigantopyramidal neurons in their motor cortex (Jacobs et al., 2018). In other words, only certain primates (those with a phylogenetically “new” M1) possess a monosynaptic connection from layer V gigantopyramidal (i.e., Betz) neurons to α‐motoneurons (Strick et al., 2021). It is presumed that this has evolved to allow fine motor control of digits, which has reached unprecedented refinement in human primates (Welniarz et al., 2017). Moreover, rodent motor cortex layer V ETPs lack specific ion channels in their soma such as the fast potassium channel Kv31b, which are present in primate layer V ETP neurons, consistent with the thin, fast‐firing spikes observed in primate but not rodent ETP neurons (Soares et al., 2017). Therefore, using rodents to study the selective vulnerability of corticospinal ETP neurons in the context of neurodegenerative diseases like ALS may result in misleading conclusions.

Assuming the morphological definitions described above, Betz cells have been identified in many primates, including ring‐tailed lemur (Campos‐Ortega & Clüver, 1969; Muñoz et al., 1999; Sherwood et al., 2003; Tigges et al., 1990, 1992; Vigneswaran et al., 2011; Vogt et al., 2005), golden lion tamarin (Jacobs et al., 2018 ), green monkey (Bucy, 1935), woolly monkey (Bucy, 1935), patas monkey (Sherwood et al., 2003), spider monkey (Bucy, 1935; Lashley & Clark, 1946), baboon (Bucy, 1935; Jacobs et al., 2018; Sherwood et al., 2004), macaques (Campos‐Ortega & Clüver, 1969; Muñoz et al., 1999; Sherwood et al., 2004, 2003; Tigges et al., 1990; Tigges, 1992; Tigges, Herndon et al., 1992; Vigneswaran et al., 2011; Vogt et al., 2005), marmoset (Bakken et al., 2021; Burman et al., 2008), orangutan (Bucy, 1935; Sherwood et al., 2004), gorilla (Sherwood et al., 2004, 2003), and chimpanzee (Bucy, 1935; Sherwood et al., 2004). Gigantopyramidal neurons are also seen in the primary motor cortex of other mammals (Badlangana et al., 2007; Brodmann, 1909; Ebinger, 1975; Groos et al., 1978; Jacobs et al., 2018, 2016; Phillips, 1959; Sherwood et al., 2003; Takeuchi & Sugita, 2001); however, their precise relationship with primate Betz cells remains to be defined. In primates, Betz cells have seen a medial and upward evolutionary shift in humans, where ∼75% are localized to the upper third of the motor strip, while, in macaques, this upper third contains only 52% of the total. Unexpectedly, nonhuman primates appear to contain comparatively more Betz cells in the arm region than humans (33% vs. 20%).

The relationship between the size of a neuronal soma, the extent of its axodendritic cell volume, or the length and caliber of its axon is complex. It is not necessarily true that large neuronal soma size equates to an extensive axodendritic tree and thus high metabolic demand. The density of afferent and efferent synaptic connectivity, firing rate, and type of neurotransmitter deployed also determine metabolic rate and neuronal soma size (Sengupta et al., 2013). For example, the axodendritic volume of an adrenergic neuron in the locus coeruleus diffusely projecting to the cerebrum (Chandler et al., 2019) is likely larger than that of the Betz cell, yet the Betz cell soma is clearly bigger than that of a catecholaminergic neuron in the locus coeruleus. Further, although the soma sizes of certain neuronal classes increase with total brain size, this is not universally true, either; a complex relationship exists, particularly in primates between brain size, increased number of cortical neurons and cell size (Herculano‐Houzel, 2012). Nor can the direct monosynaptic connectivity of primate Betz cells with α‐motoneurons be used as an argument for the large soma size of Betz cells in primates, as carnivores, particularly feliforms, do possess gigantopyramidal neurons which do not make significant monosynaptic contact to anterior horn cells and instead rely on a disynaptic route via spinal cord interneurons (Strick et al., 2021). Perhaps primate‐specific aspects of relative scaling of M1 neurons in relation to spinal cord neurons may contribute to these puzzling observations (Herculano‐Houzel et al., 2016); however, we are not aware of comparative studies on the M1 neuron/spinal cord neuron ratio that specifically stratifies animals by the presence of gigantopyramidal neurons in M1.

Therefore, until we achieve an integrated morphofunctional and metabolic characterization of the human Betz cell and its gigantopyramidal neuron counterparts in other primates and felines, we can only speculate on what drives the size of human Betz cell somata. Existing data support the idea that there is a relative increase in the size and number of gigantopyramidal neurons with a larger brain and body size, it is not an entirely linear relationship, and several species exist outside the 95% confidence interval range in this respect (Sherwood et al., 2003). Two examples of this are that of the kinkajou, which, despite weighing on average just under 3 kg, possess gigantopyramidal cells close to the mean volume of humans (Brodmann, 1909), and the patas monkey, which exhibits an unusually high Betz cell ratio despite its relatively small brain volume (Sherwood et al., 2003). Additionally, there appears to be no obvious relationship between the size of the soma of gigantopyramidal neurons and the digital dexterity of the species (Jacobs et al., 2018; Sherwood et al., 2003), with feliforms ranking low in digital dexterity yet possessing the largest gigantopyramidal cells, and primates possessing impressive dexterity yet smaller Betz cells (Heffner & Masterton, 1975). It is often believed that the large size of the Betz cells in the leg/hip area of primates can be explained by the length of the axons needed to innervate the lumbar segment of the spinal cord. However, Betz cell size in this region varies considerably and they are only the largest in the motor cortex when an average is taken (Lassek, 1948), posing the question of the nature of the smaller Betz cells in this region. By similar logic it may be expected that the giraffe would contain some of the largest gigantopyramidal neurons in the animal kingdom; however, this is not the case, and its motor cortex contains gigantopyramidal cells of comparable size to humans (Badlangana et al., 2007; Jacobs et al., 2018, 2015, 2014).

Recent comprehensive quantitative comparison of the hand/forepaw area of the primary motor cortex by Jacobs et al. (2018) on 19 mammalian species spanning 7 phylogenetic orders highlighted the remarkable variation in gigantopyramidal cell morphology across species. The authors recorded soma size and depth from the pial surface, but also dendritic volume, dendritic length, mean segment length, dendritic segment count, dendritic spine number, and dendritic spine density. Comparing superficial neurons (layer III), deep pyramidal neurons (layer V), and gigantopyramidal cells (layer Vb), they found across all species that superficial neurons contained the least morphological variation, and gigantopyramidal cells the highest, with their largest variant being the average number of primary basilar dendrites. The average number of basal dendrites across all species studied was 7.01, ranging from ∼3.7 in the clouded leopard to ∼14 in the giraffe, which confirmed previous observations (Jacobs et al., 2014). In the African lion and caracal, dendrites radiated symmetrically across the entire perikaryon, whereas, in humans, Betz cell dendrites around the soma tend to orientate themselves in the plane of the gyrus, facing toward the white matter, consistent with previous observations (Meyer, 1987).

Jacobs et al. (2018) also confirmed the observations of Brodmann, in that carnivores possess larger gigantopyramidal cells than non‐carnivores. These authors found that feliforms contained the largest cells, with an average soma of 2874 μm^2^, compared to the average 987 μm^2^ found in primate Betz cells. Within feliforms, animals in the genus *Panthera* contained the largest cells in the study with cell soma volumes 12.25 times larger than other layer V neurons (Figure 5); and although the average size of layer V neurons did not differ from that non*‐Panthera* layer V neurons, gigantopyramidal cell size was significantly larger than in other species. The dendritic volume in feliforms was also 45% higher than all other taxonomic groups. Finally, it was shown that soma size and volume were a differentiator between gigantopyramidal and other deep pyramidal neurons; however, this difference was subtle on average, with the former soma size and volume being only 1.64 and 2.3 larger, respectively. Although gigantopyramidal cell somatodendritic morphology varied considerably across species (sufficient to statistically stratify taxonomic groups), primate Betz cell morphology was remarkably uniform and consistent with previous literature in humans (Lassek, 1941; Meyer, 1987; Scheibel et al., 1974). This supports the definition adopted in this review that for the time being, stricto sensu, Betz cells are best conceptualized as the evolutionary manifestation of gigantopyramidal primary motor cortex neurons in primates. In contrast, a similar comparative study exploring the neuronal morphology in the cerebellum of carnivores, afrotherians, cetartiodactyls, and primates noted remarkable similarities across all species, especially among the magnocellular neuron of the cerebellar cortex, the Purkinje cell (Jacobs et al., 2014). The differences seen in motor cortex gigantopyramidal cell morphology across taxonomical groups may therefore represent unique functional differences associated with forebrain evolution that, in human primates, led to unique levels of fine motor control (manual dexterity, speech). Indeed, these phylogenetic considerations may be directly relevant to the observation that—to the best of our knowledge—only human primates develop spontaneously a disease pattern identifiable as ALS (Eisen et al., 1992).

The distinct “nesting,” or clustering, already observed by Betz (1874), is also a feature that varies across animals. In the giraffe and sheep brain, it is rare to find isolated gigantopyramidal neurons across layer Vb of the motor cortex; instead, they form clusters of no more than four cells (Badlangana et al., 2007; Ebinger, 1975), and this is unlike the primate brain, where single Betz cells are common. It has also been shown that cetaceans contain gigantopyramidal neurons: See studies of the sperm whale (Kojima, 1951), humpback whale (Butti et al., 2015; Hof & Van Der Gucht, 2007), and bottlenose dolphin (Butti et al., 2015; Hof et al., 2005). Interestingly, gigantopyramidal motor cortex cells in cetaceans can form clusters of up to 15 neurons (as seen in the humpback whale) and may represent a unique feature of cetartiodactyls. Jacobs et al. (2018) were unable to locate histologically defined gigantopyramidal cells in the wallaby, rabbit, or rat. Furthermore, although feliforms contained the most prominent gigantopyramidal cells, the mongoose contained very small corresponding cells—possibly attributed to differences in predatory behavior. Conversely, Brodmann stated that rabbits do have gigantopyramidal cells but these numbered among the smallest in his collection (Brodmann, 1909; Brodmann & Garey, 2006), and others have also shown the lack of discernible gigantopyramidal neurons in the wallaby motor cortex (Ashwell et al., 2005).

Many questions regarding the evolutionary trajectory of gigantopyramidal motor neurons remain. For example, their morphological prominence (at least as concerns soma size) in felines and primates may suggest functional similarities compared with other species; however, there is limited overlap in behavioral activity, digital dexterity, and the organization of their respective corticospinal tracts. Direct corticospinal connections to lower motor neurons are present only in primates, whereas non‐primates such as cats (Illert et al., 1976) or rodents (Alstermark et al., 2004; Yang & Lemon, 2003) do not possess such connections—although they may exist briefly in the developing rat brain (Maeda et al., 2016). Primates appear to use preferentially direct corticospinal connections over the propriospinal system, resulting in advanced manual dexterity and hand function (Bortoff & Strick, 1993; Heffner & Masterton, 1975; Lemon, 2008; Lemon & Griffiths, 2005; Nakajima et al., 2000). However, although we postulate that all primates have Betz cells as defined in this review, not all primates show direct cortico‐motoneuronal connections. For example, they are lacking in the marmoset (Rathelot & Strick, 2006), despite this species having Betz cells. Further, carnivores such as cats, which have fast‐conducting gigantopyramidal cells in their motor cortex, also lack direct cortico‐motoneuron connectivity (Alstermark et al., 1984, 2004; Asanuma et al., 1971; Maeda et al., 2016).

In humans, direct cortico‐α‐motoneuronal input has been shown electrophysiologically (de Noordhout et al., 1999; Palmer et al., 1992) and anatomically (Kuypers, 1964), although direct connections appear to be more common for the innervation of distal muscles and rarer in proximal muscles (de Noordhout et al., 1999; Palmer et al., 1992). Therefore, although it is tempting to use the feature of direct monosynaptic cortico‐motoneuronal connectivity as a defining feature of Betz cells, this is not supported by the available data.

## 5 MOLECULAR IDENTITY AND NEUROPHYSIOLOGY OF BETZ CELLS

### 5.1 Transcriptomic and proteomic characteristics

**FIGURE 6:**
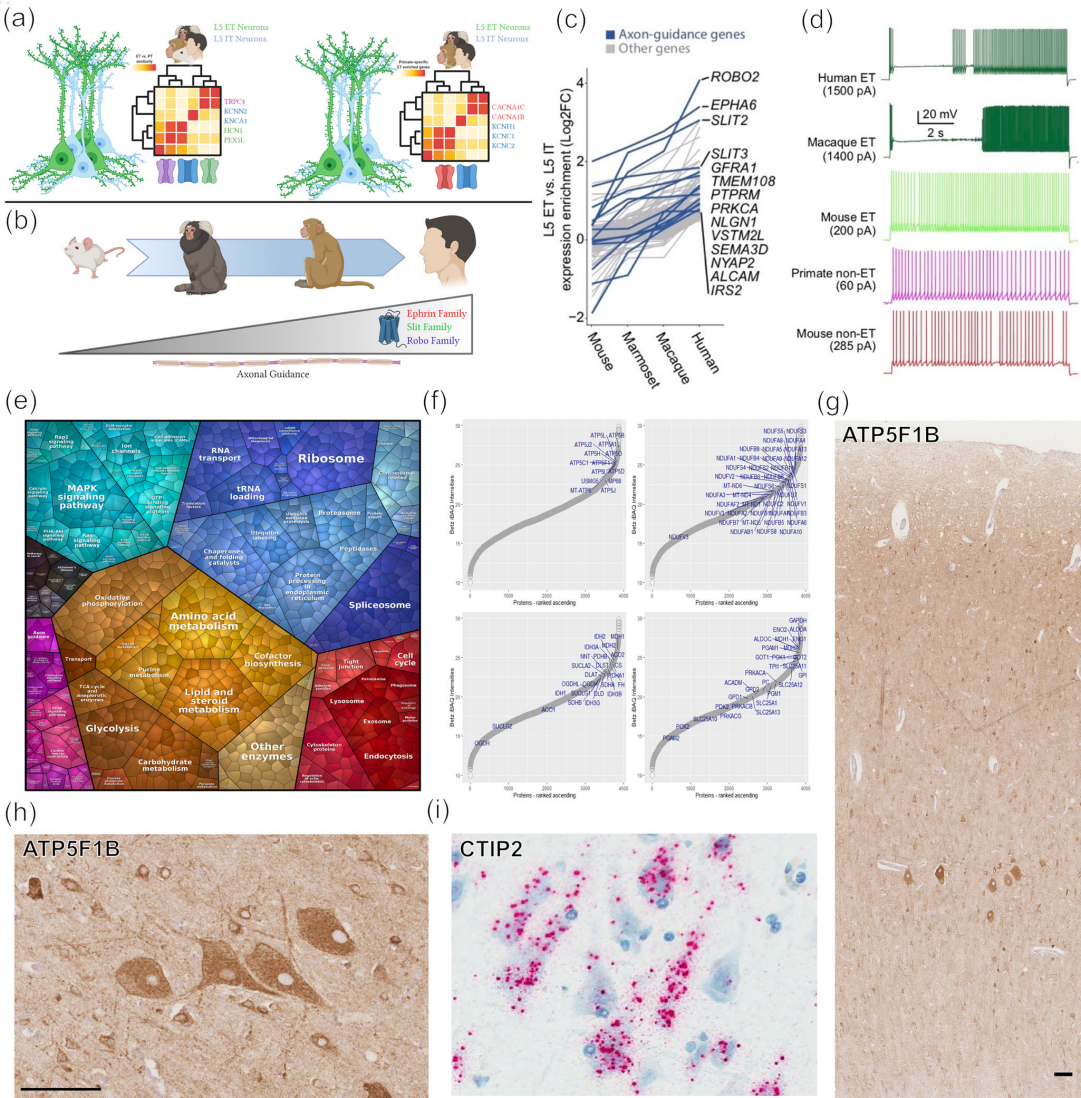
Comparative taxonomy of layer V extratelencephalic projection (ETP) neurons (including Betz cells) of the primary motor cortex. Single nucleus transcriptomic analyses (Bakken et al., 2021) revealed that ETP neurons (including Betz cells) have a higher abundance of HCN channels compared to surrounding intratelencephalic projection (ITP) neurons, with a further upregulation in calcium‐ and potassium‐voltage channels among primate ETP neurons (a). The expressions of many transcripts, particularly those involved in axonal guidance, were found to correlate with evolutionary distance to humans across species (b and c). Electrophysiologically, primate ETP neurons (i.e., Betz cells) displayed a biphasic firing pattern involving early cessation of firing followed by delayed sustained acceleration in spike frequency and magnitude, clearly distinct from rodent ETP neurons (d). Our proteomic analysis has revealed thousands of molecular species that may help define human Betz cell identity (e–i), which can be validated histologically via immunohistochemistry (e.g., *ATP5F1B*, g and h) or in situ hybridization (i, *CTIP2*). Note the importance of orthogonal tissue validation—even presumed class‐defining ETP neuron markers (e.g., *CTIP2*, to which Betz cells as a group clearly belong), may show single‐cell heterogeneity within an individual Betz‐cell cluster (i). Scale bars: 100 μm. *Source*: Panels (c and d) from Bakken et al. (2021) distributed under the terms of the CC BY 4.0 license, ET in (d) = extratelencephalic; panels (e–i), authors’ original data.

The development of cortical neurons from neural progenitors is the result of complex interactions between the tissue microenvironment and sequential expression of increasingly lineage‐restricted transcription factors, which drive and maintain differentiation post‐mitotically (Arlotta et al., 2005; Baker et al., 2018; Molyneaux et al., 2007; Shen et al., 2006). For example, it has been determined (at least in rodents) that the interaction of transcriptional programs controlled by *Fezf2*, *Ctip2*, and *Satb2* determines whether a layer V projection neuron sends its axon to extratelencephalic targets such as the spinal cord or to transcallosal targets in the contralateral hemisphere (Baker et al., 2018). According to this logic, Betz cells should express *CTIP2*, as they are considered quintessential ETP neurons. Preliminary data suggest that this is indeed the case (Figure 6i); however, expression within a Betz cell cluster may not be uniform, potentially indicating the existence of Betz cells with intratelencephalic targets (perhaps to the matching Betz cell cluster in the contralateral hemisphere; however, to date, there is no evidence for this; Catsman‐Berrevoets et al., 1980). Functionally, such within‐class molecular heterogeneity would not be surprising and has been demonstrated for other morphologically homogenous neuronal classes, such as cerebellar Purkinje cells (Lin et al., 2020). Attempts to define a specific molecular identity of Betz cells that distinguishes them from other pyramidal projection neurons in the primary motor cortex have ranged from classical immunohistochemistry to unbiased proteomics, to single‐nuclei transcriptomic and epigenomic investigations (Bakken et al., 2021; Davis et al., 2019; Network, 2021; Szocsics et al., 2021). So far, no single molecular criterion for the Betz cell class in primates has been defined; however, a constellation of molecular markers is being elucidated that refines their position in the emerging taxonomy of human neurons (Bakken et al., 2021; Network, 2021; Pineda et al., 2021). The salient findings, methods, and applied terminologies of these unbiased studies are summarized in Table 1.

In brief, despite variations in terminology and methodology, a Betz cell signature is emerging that shows some overlap in transcriptional signatures with other layer V pyramidal neurons. For example, Bakken et al. (2021) identified one macaque and two human molecular clusters with neurons that contained Betz cell morphology; their human cluster *FEZF2 CSN1S1* contained only layer V neurons, while the other human cluster *FEZF2 ASGR2* contained neurons from layers V and III (Figure 6). These clusters aligned with PATCH‐Seq analysis of macaque layer V ETP neurons and human layer V ETP neurons that were noted as being Betz cells, transcriptomic findings which were then validated via in situ hybridization. These clusters showed an enrichment of genes associated with axonal guidance—such as those from the Ephrin, Slit, and Robo families (Bakken et al., 2021). This is consistent with findings that corticospinal tract axon guidance toward the internal capsule involves receptors and ligands for Robo and Slit, and mice lacking one or both these receptors result in a ventrally displaced internal capsule with axons that aberrantly cross the midline into the ventral telencephalon (Bagri et al., 2002; López‐Bendito et al., 2007). Bakken et al. (2021) postulated that the Ephrin, Slit, and Robo families could represent genes that regulate cortico‐motoneuronal connections which allow for increasing dexterous fine motor control across species and may provide clues as to why Betz cells in most primates directly contact α‐motoneurons in addition to interneurons, which is not the case in rodents and felines. However, the expression of these transcripts is also present in non‐Betz layer V ETPs. Although these data refine the taxonomic position of the putative “Betz cell class,” they leave open the question of within‐class heterogeneity. For example, is the topographical organization and functional refinement of Betz cells involved in human hand/finger control reflected in distinct molecular features compared with those Betz cell clusters controlling the feet? Data from rodents suggest that within‐class molecular heterogeneity of layer V corticospinal projection neurons exists, depending on their axonal target region (forelimb vs. hindlimb, Golan et al., 2021; Sahni, Itoh et al., 2021; Sahni, Shnider et al., 2021). It is therefore conceivable that similar within‐class heterogeneity may exist in human Betz cells. In fact, electrophysiology studies of the domestic cat have revealed different subpopulations of giant pyramidal neurons containing two different transient voltage‐gated potassium channels which prep the giant neurons for repolarization and therefore allowing for repetitive firing, albeit at different speeds (Spain, Schwindt, & Crill, 1991; Spain, Schwindt, Crill, Spampinato et al., 1991). Further investigations of serotonin stimuli on these two groups of giant pyramidal neurons led the authors to speculate that there exist two populations of giant pyramidal neurons; one fast‐firing, the other slow‐firing (Schwindt et al., 1992; Spain, 1994). However, these studies did not provide morphological descriptions making it difficult to discern if giant pyramidal neurons were patched accurately. The concept of within‐class heterogeneity in human Betz cells has indeed been suggested by a recent study (Pineda et al., 2021). In this snRNA‐seq study of ∼380,000 human motor cortex nuclei from 64 individuals, the authors successfully annotated 46 transcriptionally distinct cell populations. They identified four subtypes of *FEZF2/CRYM*+ layer Vb neurons, of which the authors denoted clusters *BCL11B/EYA4* and *BCL11B/THSD4* as Betz cells, emphasized by their increased expression of both *NEFH* and *POU3F1* (Table 1). Finally, it is often assumed, but not proven, that in highly polarized cells such as Betz cells, the transcriptome determined from the cell nucleus is identical to that derived from presynaptic axonal compartments. It is likely, but technically virtually impossible to prove, that a more complete picture of human Betz cell identity and functional state would emerge from the integration of nuclear and distal presynaptic transcriptomes. The latter is likely to reveal mechanisms that explain (and maintain) target specificity; it stands to reason that the integrated transcriptome of a Betz cell that projects to brainstem motor neurons is distinct to that of a Betz cell innervating the lumbar spinal cord.

**FIGURE 7:**
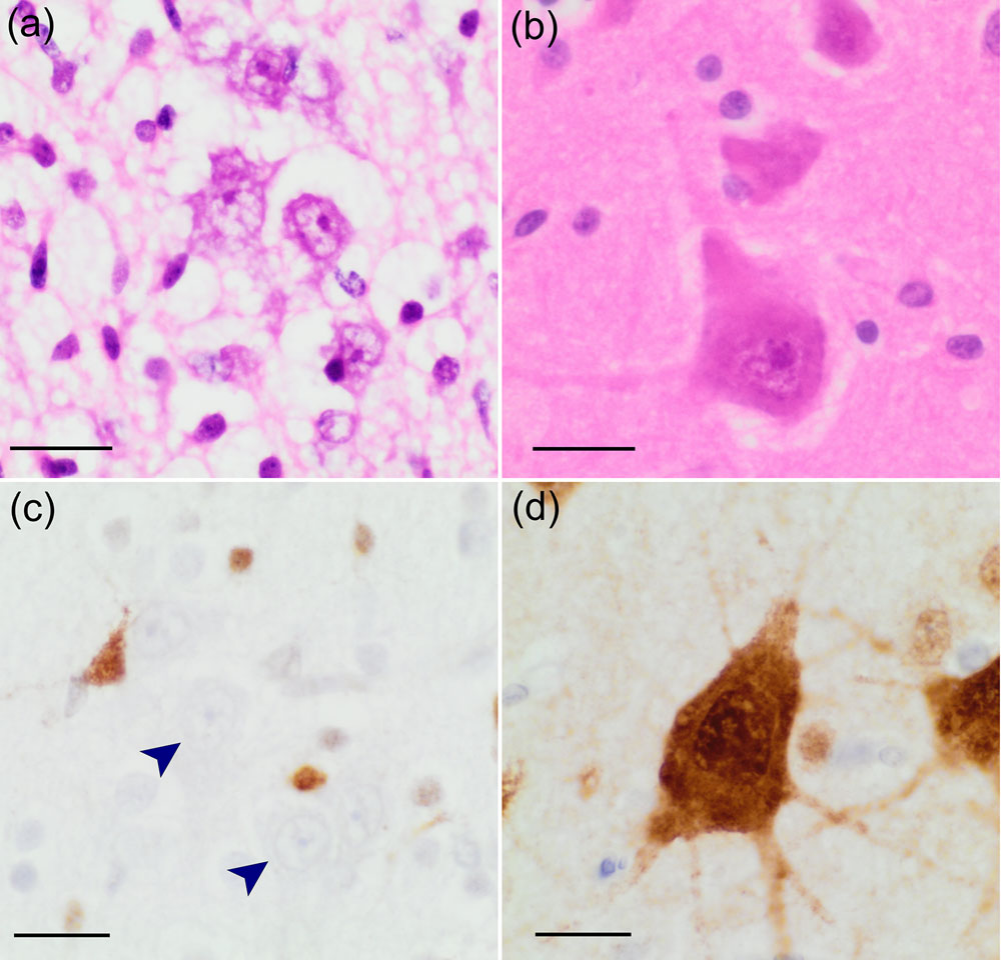
Developing human Betz cells. Characteristic clustering of the “nests of Betz” can be observed in the fetal brain (a, 29th gestational week); however, Betz cells do not express the neuronal marker NeuN (FOX‐3) at this stage (c, arrowheads, serial section to (a)), compared to adjacent smaller neurons. Postnatal Betz cells at 2 months of age clearly demonstrate a pyramidal shape (b) with numerous circumferential dendrites (d); at this stage, strong nucleocytoplasmic NeuN expression is present as a surrogate marker of emerging neuronal maturity (d). Note that vacuolation of neuropil in (a) is due to postmortem artifact of this immature brain. Scale bars: 20 μm.

At the protein level, there are surprisingly few studies of targeted (immunohistochemical) investigations of Betz cells. They are known to display abundant expression of distinct types of neurofilament, such as those detected by antibody SMI 311 against non‐phosphorylated neurofilaments (Ulfig et al., 1998), which delineate the soma and typical circumferential dendritic pattern (Figure 4c). Mature (but not first and second trimester) Betz cells react with NeuN antibody (Figures 4b and 7d). Surprisingly, Betz cells seem to express collagen 17 (Seppänen et al., 2007), a transmembrane protein thought to be involved in synaptic plasticity (Claudepierre et al., 2005; Franzke et al., 2005). Human Betz cells also contain hnRNP‐A1, the target for autoimmune IgG antibodies in human T‐lymphotropic virus type 1‐associated myelopathy/tropical spastic paraparesis (Levin et al., 2002). Like other pyramidal cells, Betz cells are surrounded by an extracellular matrix of chondroitin sulfate proteoglycan‐immunoreactive perineuronal nets (Hausen et al., 1996). Finally, the soma of human Betz cells receives a dense array of synapses from parvalbumin‐expressing interneurons (Szocsics et al., 2021), consistent with the importance of inhibitory control required for the execution of finely tuned corticospinal volitional movement.

We have used an unbiased approach to define the proteomic signature of the neuronal soma of mature human Betz cells, using laser‐capture microdissection to isolate Betz cells from adjacent cellular components of the human motor cortex (Davis et al., 2019). This proof‐of‐principle study allowed us to classify more than 3000 proteins from as few as 100 pooled magnocellular neurons of the human brain. Immunohistochemical back‐mapping of proteomic hits on tissue sections confirmed the power of this approach and allowed us to identify novel histological markers that are enriched in Betz cells versus surrounding layer V pyramidal neurons, such as ATP5F1B (Figure 6g,h), which is a subunit of mitochondrial ATP synthase. ATP5F1B is just one example of many proteins involved in neuronal energy metabolism that seem to be particularly abundant in Betz cells, perhaps reflecting the metabolic demand associated with their unique electrophysiological properties (Figure 6d) and very long axons. We anticipate that the deployment of novel techniques for in situ transcriptomics on tissue sections with spatial mapping of transcripts to nuclei or cell bodies, or mass spectrometric imaging of motor cortex sections, will yield further molecular data concerning the relationship of Betz with non‐Betz pyramidal neurons in the human primary motor cortex.

### 5.2 Electrophysiology and neurotransmitter chemistry

A comprehensive electrophysiological characterization of human Betz cells remains elusive, as most electrophysiological studies of gigantopyramidal neurons of the primary motor cortex have been performed in the domestic cat (Crawford & Curtis, 1966; Phillips, 1956, 1959; Schwindt et al., 1988). The aforementioned study (Bakken et al., 2021) revealed that ETP neurons (in the mouse, macaque, and human) display an increase in firing rate after an initial depolarizing stimulus, unlike intratelencephalic projection neurons which reduce their firing rate to a steady state after stimulation (Figure 6d). Crucially, however, the authors identified a biphasic firing pattern that appears to be unique to Betz cells in the macaque and human motor cortex (Figure 6d). This cessation in firing after an initial stimulation resulted in a delayed yet prolonged period of dramatic fast‐firing action potentials. This discharge pattern may be used to identify gigantopyramidal neurons in area 4 (Adrian & Moruzzi, 1939). Fast‐firing neurons in area 4 (assumed to include gigantopyramidal neurons) activate 40–100 ms prior to the generation of movement (Scheibel et al., 1974, 1977). This trigger is thought to partially inhibit extensor muscles and primes flexor muscle tone prior to the recruitment of neighboring layer V slow‐firing non‐gigantopyramidal neurons to facilitate motor tasks (Scheibel et al., 1974). It has been proposed that gigantopyramidal neurons do not fire but remain silent during muscle contraction and may briefly fire to relax muscles during posture control (Scheibel et al., 1974, 1977; Tigges et al., 1990). Others have suggested that within feliforms, gigantopyramidal neurons are responsible for muscle contraction velocity and power in type IIb muscle fibers (Jacobs et al., 2018). Furthermore, in lower limb movement, they appear to play a major role in controlling antigravity muscles during motion and posture, while, in upper limb movement, they may regulate the fine motor skills needed for precise control of the wrist, hand, and digits (Jacobs et al., 2018; Lemon, 2008; Scheibel et al., 1974).

In felines, corticocortical input from Brodmann area 2 synapses with layers II and III neurons of area 4; and in turn, layer III neurons send axon collaterals to the dendrites of layer V neurons, including gigantopyramidal neurons (Kaneko et al., 1994). Interestingly, the axon collaterals of gigantopyramidal neurons have been shown to provide both inhibitory and excitatory responses to neighboring neurons (Suzuki & Tukahara, 1963). Staining of this axon collateral network highlights dense neurites surrounding non‐gigantopyramidal somata (Landry et al., 1980). Gigantopyramidal neuron collaterals exhibit an “all or nothing” response (Phillips, 1956) and monosynaptically target dendrites to induce an excitatory effect. Gigantopyramidal neuron collaterals have also been shown to produce an inhibitory response (via recurrent inhibition) to adjacent neurons (Phillips, 1956; Takahashi et al., 1967). Suzuki and Tukahara (1963) proposed various explanations to this dual‐homeostatic control; most convincingly was the existence of a single inhibitory interneuron that gigantopyramidal cell collaterals synapse to, allowing for the inhibition of adjoining gigantopyramidal and non‐gigantopyramidal neurons. Later, an ultrastructural study of degenerating axons following lesion induction of the monkey motor cortex (species not specified) showed large inhibitory neurons synapsing with Betz somata and proximal dendrites (Gatter, Sloper, et al., 1978). The authors argued that this belonged to inhibitory basket cells, and although their axon terminals did not represent all axosomatic synapses on the Betz cell, they did represent a considerable proportion, highlighting their potentially critical role in pericolumnar inhibition. Basket cells, stellate cells, or other inhibitory interneurons may be responsible for the indirect inhibitory effect of Betz cell collaterals on neighboring non‐Betz pyramidal tract neurons (Gatter, Powell, et al., 1978). The importance of inhibition in the preparation and execution of fine volitional movement is increasingly becoming clear; specific intracortical collaterals are likely mediators of this sophisticated process that is ultimately executed by coordinated firing of Betz‐ and non‐Betz ETP neurons (Ebbesen & Brecht, 2017; Stefanis & Jasper, 1964). However, the cited studies span decades of research, diverse species, and use historical definitions of gigantocellular neurons—in other words, a precise electrophysiological characterization of the intracortical excitatory–inhibitory microcircuitry of the human primary motor cortex remains to be defined with novel high‐resolution surface grid electrocorticography approaches.

In an immunochemical‐based study of the brain (Williams et al., 1996), it was found that glutamate receptor (GluR) subunits have varied prevalence in human Betz cells. Although only weakly immunoreactive to GluR1, GluR2/3 showed intense immunoreactivity on Betz cell soma and proximal dendrites, which was also seen in other layer V neurons (Williams et al., 1996). An in situ hybridization study was performed on the rhesus and Japanese macaques sensorimotor cortex and found similar results, although GluR1 immunoreactivity was moderate, and Betz cells were also positive for GluR5/6/7, NR2A, and NR2A/B, confirming the presence of a variety of AMPA and NMDA receptors (Muñoz et al., 1999). An antibody capable of differentiating GluR2 and GluR3 has shown that human Betz cells do not express the GluR2 subunit (Shaw & Eggett, 2000; Shaw et al., 1999). This lack of GluR2 suggests the presence of an uncommon calcium‐permeable AMPA receptor that has been shown to strengthen signal transmission (Pellegrini‐Giampietro et al., 1997) but enhances neuronal susceptibility to glutamate toxicity (Shaw & Eggett, 2000; Shaw et al., 1999).

In macaques, Betz cells also express GABAA receptors—a fast‐inhibitory bicuculline‐sensitive ionotropic receptor (Huntsman et al., 1995). Betz cells were found to express several types of GABAA subunits, including high amounts of mRNA from GABAA γ2, moderate amounts of α1, and small amounts of β2 and α5, while β1, α2, and α4 subunits could not be resolved with in situ hybridization. The positivity for α1, α5, β2, and γ2 mRNA suggests that Betz cells at least possess β2 subunit GABAA receptors and β3 subunit GABAA receptors, which has been confirmed using complementary radioactive RNA probes in macaques (Huntley, de Blas, & Jones, 1990; Huntley, de Blas, Jones, Huntsman et al., 1990). GABAA receptors have been shown to regulate plasticity in the visual cortex, but also the plasticity of the homunculus in the motor cortex of adult animals (Huntsman et al., 1995; Jacobs & Donoghue, 1991). Feline gigantopyramidal neurons have been shown to be excited by glutamate (l‐glutamate) and glutamate agonists, *N*‐methyl‐d‐aspartic acid and dl‐homocysteic acid. Acetylcholine has also been shown to excite Betz cells in monkeys and gigantopyramidal cells in cats (Crawford, 1970; Krnjevic & Phillis, 1963), while acetylcholinesterase is almost always found in human Betz cells (Mesulam & Geula, 1991). Inhibitory effects are mediated by GABA and the GABA agonist, 3‐amino‐1‐propanesulfonic acid (Crawford & Curtis, 1964). Additionally, further studies have shown that both serotonin (5‐hydroxytryptamine) and dopamine (3‐hydroxytyramine) had equally potent inhibitory effects on gigantopyramidal cells (Crawford & Curtis, 1966). A combined light and electron microscopic study demonstrated abundant synaptic input from parvalbumin‐expressing interneurons (Szocsics et al., 2021).

A comprehensive investigation of human brain integrated cyto‐ and myeloarchitectural features of cortical regions with quantitative in vitro receptor autoradiography for 17 neurotransmitter systems (Palomero‐Gallagher & Zilles, 2019) did not allow cell‐specific assignment of receptor subtypes; however, it provides an indication of layer‐specific distributions, including Vb, in which Betz cell somata are located, and layers I–III which contain their apical dendrites. All receptors in area 4 showed a unimodal distribution with the highest concentrations in layers I–III (Figure 2g). Overall, the receptor pattern in area 4 was distinct from all other isocortical areas. Evidence of cholinergic innervation was confirmed, with the demonstration of both nicotinic and muscarinic receptors in the upper layers.

In summary, it remains difficult to synthesize a clear picture concerning the repertoire of human Betz cell‐specific neurotransmitters and receptors, their quantitative relationships, and physiological effects. The systematic application of novel intraoperative electrocorticography technology and ex vivo imaging methods such as light‐sheet microscopy and mass spectrometric imaging may help resolve this.

## 6 BETZ CELLS ACROSS THE HUMAN LIFE SPAN AND IN NEUROLOGICAL DISEASE

### 6.1 Development

The lack of a unifying Betz cell nomenclature is a significant challenge when extrapolating findings from the developing brain. Marín‐Padilla (2011) performed over 4500 Golgi preparations on the primary motor cortex of 27 prenatal cases ranging from sixth week gestation to the newborn and showed that the embryonic cytoarchitectural organization of the cerebrum begins at approximately the 7‐week stage with the development of the lateral and third ventricle, choroid plexus, and an anlage of the hippocampus. At this early stage, fibers can be seen passing through the internal capsule and terminating at the subpial zone containing Cajal–Retzius neurons. At 7 weeks, cortical organization is considered functionally active, and deep neuron‐like cells from the subplate can be seen with efferent corticofugal fibers (Larroche, 1981; Marín‐Padilla, 2011). By this point, the developing brain is equivalent to the 11‐day mouse, or 22‐day cat brain (Marín‐Padilla, 2011). Motor cortex features can be recognized by the 11th week of gestational age; the first lamina (adult nomenclature: layer I) can be seen with Cajal–Retzius cells and their horizontal fibers. Migrating neuroblasts are guided by radial glia fibers and extend to the first lamina where they lose their glial attachment and form an apical dendrite that attaches to the first lamina (Marín‐Padilla, 2011). Collectively, these neuroblasts will form the pyramidal cell plate (PCP) which eventually will give rise to all the pyramidal neurons in this cortex. However, at this stage, the PCP is composed of small densely packed neuroblasts lacking dendritic spines, with smooth cell bodies giving rise to axons that have not yet reached the white matter. These PCP neurons are not yet considered functionally active (Marín‐Padilla, 2011; Mrzljak et al., 1992). Additionally, at this age, the embryonic subplate forms neurons which are the largest and most developed neurons in the brain at that time. The apical dendrites of these large neurons branch and synapse with the first lamina; their axons descend to the white matter and become corticofugal fibers and their axons form ascending collaterals that reach the first lamina and subplate. Marín‐Padilla noted that the large pyramidal neurons of the subplate and the Cajal–Retzius cells are interconnected, with both receiving corticopetal input.

By 15 weeks, the human PCP is arranged in columns with vertical cell‐free zones filled with radial glia filaments, ascending corticopetal fibers, and the descending axons of PCP neurons. Although the motor cortex is still immature, the PCP is nearly completely formed, and no additional neurons are introduced after 18 weeks of gestation. By 15 weeks, it is possible to determine the age of the neurons based on the length of their apical dendrite, as this reflects their arrival to the first lamina. Older neurons will elongate their apical dendrite to maintain their anchorage to the first lamina; however, their somatic location will not change (Marín‐Padilla, 1998; Marín‐Padilla, 1992). At this stage, there is great variety in the size of PCP cells, ranging from 30 μm for superficial neurons to 275 μm for deeper, older neurons. Deeper neurons begin to form basilar dendrites and proximal dendritic spines as they mature and become the first pyramidal cell (P1) stratum in the primary motor cortex. This P1 stratum corresponds to layer V in the postembryonic brain and is the destination of Betz cells, which are postulated to be some of the first neurons to form in this stratum. Interestingly, our observations from neuropathological practice suggest that human Betz cells are not the first neurons in layer V to react with NeuN (FOX‐3) antibody (Figure 7c), whose expression is generally associated with increased maturation of cortical neurons (Sarnat et al., 1998). Marín‐Padilla noted that some of the large neurons in P1 may become gigantopyramidal neurons, although the term Betz cell is not used. In the brain of neonates, the giant cells of Betz reach sizes of ∼1600 μm^2^, while younger neurons and neurons in other strata are around 100 μm^2^. These giant cells possess long collaterals, numerous basal dendrites, and apical dendrites with or without bifurcation. They are now strongly NeuN‐positive, which highlights these features (Figure 7d). Their full dendritic architecture contains thousands of spines (Marín‐Padilla, 1967), “countless” direct axodendritic synaptic contacts, and specific terminals from inhibitory interneurons. The apical dendrite of these large pyramidal neurons of P1 elongates from ∼25 to ∼700 μm by birth.

An earlier histological study also identified Betz cells in the neocortex of newborn humans (Conel, 1947), although, unlike Marín‐Padilla, this study claimed that Betz cells were the first and most developed cells of the motor cortex, suggesting an early stage of differentiation from other pyramidal cells. However, if NeuN expression is used as a surrogate marker of neuronal maturity, this may not be true (Figure 7c). In another historical study of the cytoarchitecture of an 18‐week‐old fetus, Boltan and Moyes (1912) observed the presence of Betz cells during the beginning of prefrontal cortical lamination. The authors mapped out the area where Betz cells occurred and found them dispersed anterior to the furrow of Rolando, resembling a primordial version of the adult area 4 (Bolton & Moyes, 1912). Furthermore, they found Betz cells bordering the fissura cinguli and noted that Betz cell development in this area was not yet complete. To explain this finding, they proposed the existence of an intermediate type of cortex residing between the Betz cell area, across the cingulate fissure, and up to the “lower lip” of the cingulate gyrus. This region may perhaps form the primitive Betz cell field described by Braak (Braak, 1976; Braak & Braak, 1976) in the adult cingulate cortex. It is tempting to speculate that Betz‐like cells in this area may be involved in differential emotional and volitional motor control of facial muscles via the facial motor nucleus, or vocalizations (Müri, 2016). Boltan and Moyes (1912) continued to note that the only discernible maturing neuron was the Betz cell in layer V in this 18‐week motor cortex; the remaining part of the cortex consisted of “neuroblasts.” Although their nuclei were uniform in size, these embryonic Betz cells were half the size of adult Betz cells and were relatively narrower and more pyramidal in shape (Figure 7b). As far as we are aware, there are no modern morphomolecular data at cell‐type‐specific resolution of the developing human primary motor cortex that would help to clarify these somewhat contradictory and anecdotal historical observations. We predict that the study of neural intermediate progenitor cells, destined for the primary motor cortex, will reveal distinct morphomolecular precursors of Betz cells, as heterogeneity in this common precursor pool of excitatory cortical neurons is increasingly recognized (Pebworth et al., 2021).

### 6.2 Normal aging

Betz cells undergo morphological alterations during aging; whether there are species‐specific patterns is not clear from the literature. The most obvious age‐related occurrence is the accumulation of cytoplasmic lipofuscin, which, although seemingly well tolerated by Betz cells (Tigges, Herndon et al., 1992; Tigges, 1992), may still displace the extent of protein synthesis performed at their endoplasmic reticulum (Mann & Yates, 1973). Another prominent feature of human aging Betz cells is the progressive loss of circumferential dendrites (Hammer et al., 1979; Scheibel et al., 1977). In the rhesus monkey, Betz cell number decline is only weakly associated with aging, barely being statistically significant (Tigges et al., 1990), which is in sharp contrast to the nature of the human Betz cell in aging (Scheibel et al., 1977). In human area 4, Scheibel et al. (1977) noted that more than 75% of Betz cells may degenerate by the eighth decade of life and suggested that Betz cells’ potential role in temporarily relaxing extensor muscles prior to movement may be progressively lost during aging, which may result in pain, stiffness, and slowed motor movements seen in the hip/lower extremities of aging humans (Scheibel et al., 1977). It should be noted that these studies do not use modern quantitative techniques, such as stereology. The relationship between features of normal aging and specific motor neuron diseases such as ALS has been explored at the structural level (Hammer et al., 1979, Figure 8b); however, molecular signatures of physiological aging of Betz cells in healthy human brains remain unknown.

### 6.3 Neurodegeneration and selective vulnerability

The concept of selective vulnerability is central to attempts to understand the pathogenesis of human neurodegenerative diseases. It postulates that specific neuronal cells and systems are preferentially vulnerable to disease processes triggered by specific genetic or environmental metabolic disturbances. This is most strikingly illustrated in monogenic Mendelian diseases, where despite widespread expression of the mutant gene product, only specific cell types degenerate and “drive” the disease phenotype. The nature of cell‐intrinsic biochemical properties that make a cell vulnerable or resistant to specific insults remains largely elusive (Fu et al., 2018). Motor neuron diseases such as ALS and HSP are defined by dysfunction and loss of pyramidal cells in the primary motor cortex and degeneration of the corticospinal tract. Both disorders show complex genetic etiologies (Kim et al., 2020; Panza et al., 2022); in contrast to HSP, ALS is characterized by additional loss of α‐motoneurons in the brainstem and anterior horns of the spinal cord, which is generally absent in HSP. In both disorders—which to our knowledge are distinctly human neurological conditions—early and primary degeneration of Betz cells is postulated to play a major role. This is in contrast to most (Fischer et al., 2004; Gordon et al., 2018; Magrané et al., 2014; Sharma et al., 2016) but not all (Genc et al., 2020; Marques et al., 2021) studies in rodent models of ALS, in which α‐motoneurons may degenerate before neurons in the motor cortex. However, the corticospinal connectivity in rodents is very distinct from that of humans. The cortical “dying‐forward” hypothesis of ALS has been derived from human studies, which identify cortical dysfunction as the primary pathophysiological event prior to spinal motor neuron degeneration, potentially mediated by an anterograde, trans‐synaptic, mechanism of multiple possible etiologies (Dharmadasa, 2021; Eisen, 2021). This includes the documentation of cortical hyperexcitability occurring early in the progression of ALS patients (Menon et al., 2015), even in the presymptomatic phase (Vucic et al., 2008). This idea is supported by neuropathological considerations of corticofugal connectivity mediating the spread of abnormal proteoforms of TDP‐43 (transactive response DNA‐binding protein 43 kDa), whose presence in a stereotypical pattern characterizes ∼97% of ALS brains (Braak et al., 2013). Indeed, there are some data that suggest cortical hyperexcitability is directly linked with the expression of pathological forms of TDP‐43 (Weskamp et al., 2020).

Although most neuropathologists would expect to identify neuronal loss in the motor cortex of ALS patients to at least some extent, pathological studies of postmortem cases offer differing perspectives on the specific nature and severity to which this occurs. Two stereological studies found no difference in mean neuronal number, perikaryon volume, mean neuronal nuclear volume, total perikaryon volume, or total nuclear volume between ALS and control motor cortex (Gredal et al., 2000; Toft et al., 2005), despite neuronal loss being suggested in vivo by magnetic resonance spectroscopy (Pioro et al., 2000). This led to speculation that motor cortex dysfunction in ALS is caused by regional metabolic changes, rather than microanatomical alterations. However, these studies preceded the discovery of TDP‐43 as a marker of ALS pathology and were not designed to assess Betz cell alterations specifically. Ultrastructural changes and loss of layer V neurons, including Betz cells, are acknowledged in most cases of ALS (Coan & Mitchell, 2015; Hammer et al., 1979; Kiernan & Hudson, 1991; Nihei et al., 1993); the discrepancy is probably due to sampling methods and the difficulty in quantifying the loss of neurons that constitute only a small proportion of the total. Interestingly, there appears to be a significant correlation between the level of neuronal loss, including Betz cells, between areas 4 and 3 (Mochizuki et al., 2011), suggesting a degree of regional interdependence.

Morphometric investigations have provided evidence of the preferential vulnerability of “large” corticospinal tract axons in patients with ALS regardless of their dominant neurological signs (Oyanagi et al., 1995; Riku et al., 2014). Although the definition of what constitutes a “large” corticospinal axon varies among studies (1–14 μm), it is presumed that the largest axons in the CST originate from Betz cells. If true, the preferential vulnerability of large axons in the CST of ALS patients may reflect the selective vulnerability of Betz cells; however, the pattern of TDP‐43 neuropathology indicates that other layer III and V pyramidal neurons are also affected by disease. Which cells initiate the degenerative process is difficult to establish in postmortem studies that reflect the terminal phase of the disease. A definitive analysis of any correlation between the loss of the largest axons in the human CST and Betz cell degeneration will depend on the identification of axoplasmic Betz cell‐specific markers or tracers.

**FIGURE 8:**
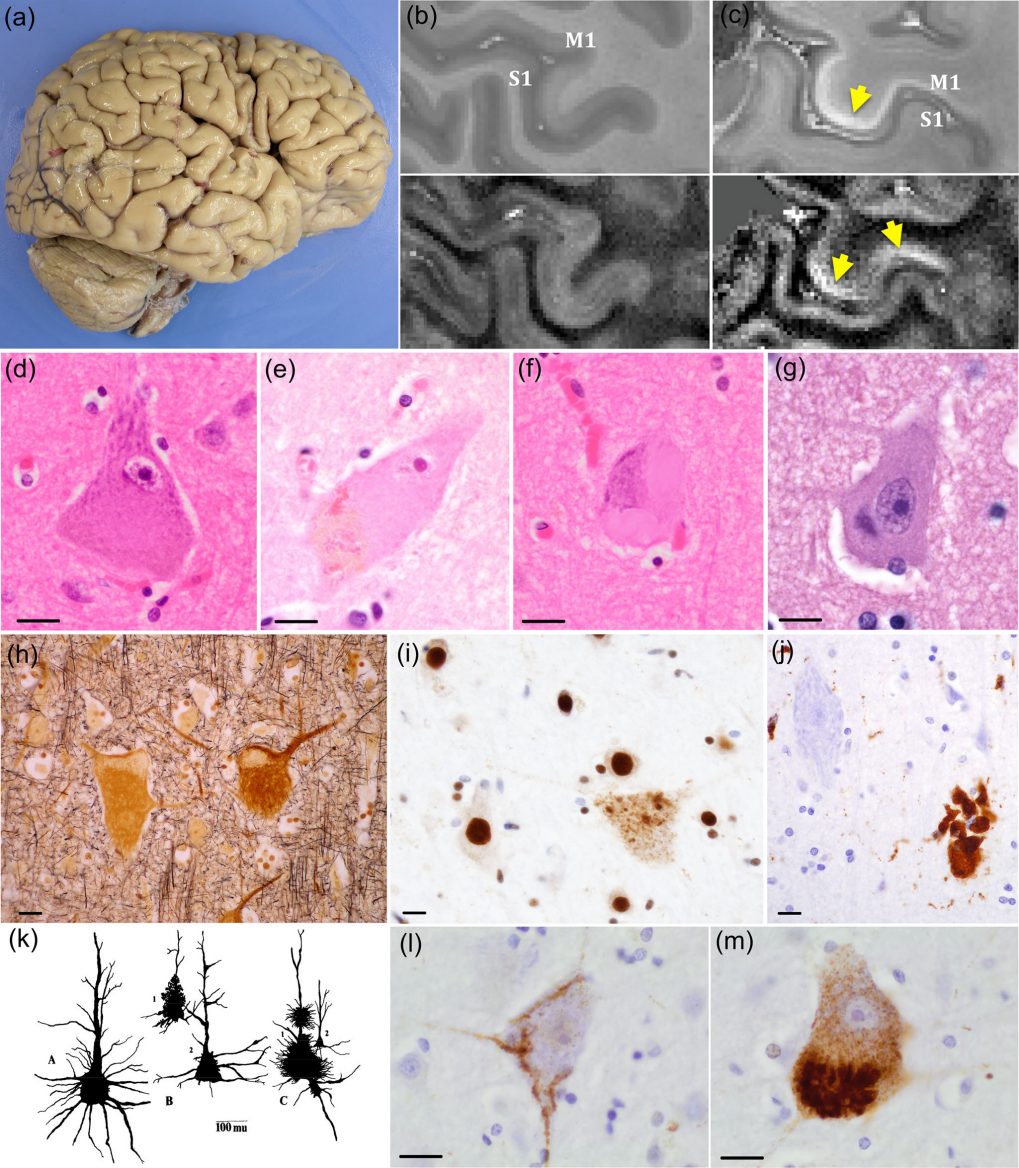
The Betz cell in neurodegeneration. Macroscopic atrophy of the primary motor cortex in amyotrophic lateral sclerosis (ALS) (a) is rare and generally restricted to those who clinically presented with the primary lateral sclerosis phenotype of ALS. Most cases of classical ALS show only subtle changes in area 4, best visualized with *R* ^2^* and susceptibility magnetic resonance imaging (MRI) (b and c). Note increased signal in the “hand knob” of area 4 in ALS (c, arrows) compared with healthy cortex (b). Such severe atrophy and signal change in (a–c) are histologically associated with neuronal loss that involves Betz cells but also other pyramidal cells in layers V and III. Some cytomorphological features visible on routine hematoxylin and eosin‐stained sections may be indicative of specific forms of ALS: In sporadic ALS (d and e), Betz cells may show chromatolysis only (d) or accumulation of eosinophilic granular material (Bunina bodies, structures of uncertain origin containing cystatin C, and transferrin) in chromatolytic neurons (e); these features are generally associated with transactive response DNA‐binding protein 43 kDa (TDP‐43) pathology. Large amorphous hyaline aggregates in the cytoplasm of Betz cells (f) are seen in *SOD1 I114T* ALS, which is not associated with TDP‐43 pathology. Basophilic cytoplasmic inclusions (g) characterize ALS with *FUS* mutations (that are also TDP‐43‐negative). Bielschowsky silver impregnation may show increased fibrillarity in ALS Betz cell cytoplasm (h). The most common form of ALS is linked to TDP‐43 mislocalization from the nucleus (i, left, normal Betz cell) to the cytoplasm (i, right, granular cytoplasmic reaction product). Active neuronophagia of Betz cells may be observed (j, CD68). Historic Golgi studies highlight astrocytic fibrosis around Betz cell somata in ALS (k). Other neurodegenerative disorders, notably tauopathies, may also show selective Betz cell pathology (l and m; globular tauopathy, AT8). Scale bars: 50 μm. *Source*: Reproduced and modified with permission from the publishers: (a) Nolan et al. (2021), (b and c) Pallebage‐Gamarallage et al. (2018), (d) Baumer et al. (2010), and (k) Hammer et al. (1979).

TDP‐43 is a DNA/RNA‐binding protein involved in several aspects of RNA metabolism, including transcriptional repression, splicing, and translational regulation (Kuo et al., 2009), and is highly expressed within the Betz cell nucleus (Figure 8i). Notably, although its expression is highly conserved across mammalian species, the specific splice targets of TDP‐43 are not (Polymenidou et al., 2011; Tollervey et al., 2011). Neuronal and glial cytoplasmic inclusions immunoreactive for mislocalized TDP‐43 are the primary neuropathological characteristic of the majority of ALS cases (Neumann et al., 2006). Despite the loss of nuclear TDP‐43, Betz cells only rarely accumulate the characteristic cytoplasmic aggregation of insoluble, hyperphosphorylated TDP‐43 associated with ALS (Braak et al., 2017, Figure 8i) despite other smaller, neighboring pyramidal cells being affected. There is also no correlation between Betz cell loss and the severity of phosphorylated TDP‐43 pathology in motor cortex gray matter (Fatima et al., 2015), although the extent of TDP‐43 pathological severity varies significantly between patients (Nolan et al., 2020). However, a recent study described early mitochondrial and nuclear membrane‐related defects arising from cytoplasmic TDP‐43 mislocalization in both human Betz cells and a mouse corticospinal layer V reporter line (Gautam et al., 2019). Nodular microgliosis and neuronophagia of layer V neurons, most likely targeting Betz cells, are also occasionally seen (Nolan et al., 2020, Figure 8j). These observations raise interesting questions regarding any potential mechanisms for the selective vulnerability of Betz cells in ALS. Are they more vulnerable to the increased toxicity of soluble TDP‐43, perhaps because of the higher importance of RNA/protein homeostasis related to their larger size or metabolic activity? Do they have a reduced capacity to package abnormal proteoforms into potentially neuroprotective aggregates compared to neighboring neurons and oligodendrocytes? Genetic clues, implying the importance of the integrity of the endosomal‐lysosomal and ubiquitin‐proteasomal systems for preserved motor function may support this idea (Fu et al., 2018).

Several studies have identified dendritic degeneration as a prominent feature of Betz cell pathology in ALS (Genc et al., 2017; Hammer et al., 1979; Jara et al., 2012; Udaka et al., 1986). In a recent study, Genç et al. (2017) reported profound apical dendrite degeneration in both familial and sporadic ALS, including extensive dendritic vacuolation and cytoarchitectural disintegration which appeared to be most severe distally from the soma. The specific morphologies and molecular compositions of protein aggregates affecting Betz cells have not been systematically explored. However, distinct types have been recognized, often representing proteins known to be affected by mutations in their respective genes (Baumer et al., 2010; Nolan et al., 2020, Figure 8). Betz cell involvement is particularly severe in the primary lateral sclerosis variant of ALS (Mackenzie, 2020), characterized by minimal involvement of α‐motoneurons (Pringle et al., 1992), and in HSP (Fink, 2013; Seidel et al., 2009). One study also found a reduction in Betz cell number in two cases of juvenile spinal muscular atrophy (Araki et al., 2003, a disease primarily affecting α‐motoneurons), while they seem to be unaffected in the adult form (Huang et al., 1983). Finally, there are reports of prominent Betz cell loss in spinocerebellar ataxia type 2 (SCA2, Hoche et al., 2011) and type 6 (SCA6, Gierga et al., 2009; Kang et al., 2017).

There is also evidence of Betz cell involvement in several age‐related neurodegenerative diseases that do not manifest primarily as a motor neuron disorder (Table 2). In frontotemporal lobar degeneration (FTLD) with microtubule protein tau aggregation (FTLD‐Tau), Betz cells degenerate and are replaced by clusters of macrophages containing lipofuscin and also occasionally contain intracellular accumulations of tau (Pick bodies, Tsuchiya et al., 2006). More prominent degeneration and intracellular tau accumulation of Betz cells are seen in the corticobasal degeneration variant of FTLD‐Tau (Tsuchiya et al., 2005) and rare forms of progressive supranuclear palsy. The most striking tau pathology can be seen in FTLD‐Tau with globular aggregates (Figure 8l,m). Involvement of Betz cells in the prototypical neurodegenerative synucleinopathy, Lewy body Parkinson's disease, is rare, affecting 1.1% of Betz cells (Wakabayashi et al., 2003) but relatively common in multiple system atrophy (Tsuchiya et al., 2000). There have also been reports of Betz cell pathology in late‐infantile Friedreich's ataxia (Koeppen & Mazurkiewicz, 2013). In lathyrism, a systemic disease caused by eating seeds of the *Lathyrus*, it is debated if Betz cell loss is the cause of spastic paraparesis (Giménez‐Roldán et al., 2019). Given the functionality of Betz cells and the primarily non‐motor symptomatology of most of these disorders, it seems likely that these phenomena are late‐stage manifestations perhaps driven by the transsynaptic spread of abnormal proteoforms via afferents from prefrontal areas that are the primary targets of neurodegeneration in this context.

**TABLE 2:** Betz cell Neuropathology.

Neurological disease/disorder	Summary of Betz cell neuropathology	Reference
Amyotrophic lateral sclerosis	Atrophy and/or complete depletion of Betz cells/dendritic degradation	Genc et al. (2017)
Corticobasal degeneration	Loss of Betz cells with prominent astrocytosis/presence of ballooned neurons	Tsuchiya et al. (2005)
Down's syndrome	Increase in apical dendritic spines/dendritic spine abnormalities	Marín‐Padilla (1976)
Friedreich's Ataxia	Atrophy and/or complete depletion of Betz cells	Koeppen and Mazurkiewicz (2013)
Frontotemporal dementia (Pick's disease)	Loss of Betz cells with prominent astrocytosis/presence of ballooned neurons (Pick bodies)	Tsuchiya et al. (2006)
HAM/TSP	Betz cell axonal damage	Levin et al. (2002)
Hereditary spastic paraplegia	Betz cell atrophy	Meyyazhagan and Orlacchio (2022)
Juvenile spinal muscular atrophy	Loss of Betz cells with prominent astroctosis	Araki et al. (2003)
Late‐infantile galactosialidosis	Loss of Betz cells with prominent astrocytosis/presence of ballooned neurons with accumulating material	Oyanagi et al. (1991)
Lathyrism	Conflicting reports on Betz cell atrophy	Giménez‐Roldán et al. (2019)
Multiple systems atrophy	Atrophy and/or complete depletion of Betz cells with/without astrocytosis	Tsuchiya et al. (2000)
Parkinson's disease	Presence of ballooned neurons with α‐synuclein (Lewy bodies)	Wakabayashi et al. (2003)
Primary lateral sclerosis	Atrophy and/or complete depletion of Betz cells	Rollins et al. (2009)
Spinocerebellar ataxia	Atrophy and/or complete depletion of Betz cells	Hoche et al. (2011)

*Note*: Summary of human Betz cell abnormalities in various neurodegenerative disorders.

Abbreviation: HAM/TSP, human T‐lymphotropic virus type 1 (HTLV‐1)‐associated myelopathy/tropical spastic paraparesis.

## 7 CONCLUSIONS

Betz cells—here defined as gigantopyramidal ETP neurons of layer Vb of the primary motor cortex in primates—clearly belong to the group of ETP neurons seen in other species in homologous cortical layers. Despite their morphological prominence in the human neocortex, relatively little data exist about their developmental origin and transcriptional program driving their positional and functional identity in the motor circuit. In our view, their unique electrophysiological characteristics, monosynaptic connectivity to bulbar and spinal α‐motoneurons, clustering within “nests of Betz”, and peculiar dendritic architecture suggest that they may represent a distinct subtype of layer V pyramidal neurons. In humans, their evolution and integration into the motor circuitry may be linked to the emergence of unparalleled manual dexterity and speech. Single‐nucleus and pooled Betz cell analyses from the human brain are beginning to identify molecular markers that distinguish Betz cells from surrounding layer V pyramidal neurons. However, currently available data are insufficient to formulate a categorical view on whether a unique Betz cell signature exists, or, if indeed all gigantopyramidal neurons in a human Betz cell nest are molecularly homogenous. Biochemical heterogeneity of prima facie morphologically identical neurons is well established.

We are confident that novel technologies and prospective brain tissue collection strategies will allow us to find answers to some of the most obvious questions relating to Betz cell taxonomy across the human life span and evolutionary distance (see Table 3): Is there a specific location within the cortical plate where Betz cells arise? What are the earliest molecular markers that distinguish them from other layer V ETP neurons of the primary motor cortex? Are there molecular characteristics that distinguish Betz cell nests innervating the legs from those innervating the hands? Is there Betz cell molecular heterogeneity within nests? Do Betz cell axons arising from neurons within the newly proposed “inter‐effector” regions of M1 (Gordon et al., 2023) project to intratelencephalic targets of the cingulo‐opercular network? What molecular/anatomical similarities are there between primate Betz cells and gigantopyramidal motor neurons of other mammals? Multidisciplinary research should be able to answer these questions; however, this would require prospective collection of tissue, as standard dissection protocols do not accommodate the distinct mediolateral trajectory of the primary motor cortex and requirements for multiomic analysis at cell‐type‐specific resolution. Even if the motor cortex is sampled appropriately, this may not reveal the full molecular architecture of the Betz cell—as the large majority of their functionally active biomolecules reside outside their nucleus and soma.

**TABLE 3:** Unanswered/remaining questions regarding Betz cells.

The Betz cell as a morphofunctional unit of the human brain—remaining questions
Can further single‐cell and spatial analyses of the adult human motor cortex refine the taxonomic position of the Betz cell within the group of ETP layer V projection neurons of the primary motor cortex? Are all Betz cells created equal? Is there heterogeneity of Betz cell clusters across the functional zones of the primary motor cortex (e.g., hand vs. foot)? Is there Betz cell heterogeneity within a “nest” of Betz cells? If the recently postulated “integrate‐isolate” model of M1 organization is correct, does this mean that Betz cells located in the “inter‐effector” zones project axon collaterals to ITP targets? Does the current (methodological) restriction of single‐nuclei transcriptomic analyses prevent us from uncovering the “true” molecular signature of the Betz cell class in the human motor cortex? Betz cells give rise to some of the longest axons in the human central nervous system and much of their topographical and functional transcriptomic signature may be encoded in their axonal terminals (via localized mRNA expression). What transcriptional regulators drive Betz cell differentiation and topographical destiny during human corticogenesis? What is the molecular relationship of Betz cells with other relatively unique neuronal classes of the human brain; specifically, the “von Economo” neurons of the fronto‐insular and anterior limbic areas, which are also deemed selectively vulnerable to diseases of the ALS‐FTLD group of degenerative brain disorders? Is the physiological expression pattern or concentration of transcripts of genes implicated in monogenetic forms of ALS or HSP different in Betz cells vs. other L5 ETP or ITP projection neurons in M1? If so, does this information help to resolve the question of “selective vulnerability” to ALS and HSP? Does the loss of nuclear TDP‐43 expression in Betz cells lead to distinct splicing defects compared with other neuronal classes in the human CNS , providing a mechanistic insight into selective vulnerability?

*Note*: We use “Betz cell” here as an operational term in the context of this manuscript. Any answers to the questions posed above will inform us about a better terminology for this type of cell, which may replace the eponymous designation of these neurons.

Abbreviations: ALS, amyotrophic lateral sclerosis; ETP, extratelencephalic projection; FTLD, frontotemporal lobar degeneration; HSP, hereditary spastic paraplegia; ITP, intratelencephalic projection; TDP‐43, transactive response DNA‐binding protein 43 kDa.

Finally, we argue that these pursuits are not merely academic exercises. We believe that vulnerability to ALS may be intrinsically linked to the evolution of human‐specific levels of volitional fine motor control, which must find its basis in the cytoarchitecture and connectivity of the human primary motor cortex, in which Betz cells fulfill a crucial role. In other words, the study of human Betz cell physiology may give us clues to mechanisms of selective vulnerability in ALS, and thus perhaps novel targets for treatment.

## AUTHOR CONTRIBUTIONS

Olaf Ansorge conceived the review, structure, content, selection of figures and revised the final draft. Matthew Nolan and Connor Scott contributed equally to the initial drafts. Olaf Ansorge, Connor Scott, Matthew Nolan, and Patrick. R. Hof edited and agreed on the final version. We are particularly grateful to Roger Lemon (Department of Clinical and Movement Sciences, Queen Square Institute of Neurology, UCL, London, WC1N 3BG, UK) who advised us on the drafts of this manuscript.

## CONFLICT OF INTEREST STATEMENT

We declare no conflicts of interest

### PEER REVIEW

The peer review history for this article is available at https://publons.com/publon/10.1002/cne.25567.

## Data Availability

Data sharing is not applicable to this article as no new data were created or analyzed in this study.
